# Identification of a Retroelement from the Resurrection Plant *Boea hygrometrica* That Confers Osmotic and Alkaline Tolerance in *Arabidopsis thaliana*

**DOI:** 10.1371/journal.pone.0098098

**Published:** 2014-05-22

**Authors:** Yan Zhao, Tao Xu, Chun-Ying Shen, Guang-Hui Xu, Shi-Xuan Chen, Li-Zhen Song, Mei-Jing Li, Li-Li Wang, Yan Zhu, Wei-Tao Lv, Zhi-Zhong Gong, Chun-Ming Liu, Xin Deng

**Affiliations:** 1 Key Laboratory of Plant Resources, Institute of Botany, Chinese Academy of Sciences, Beijing, China; 2 Key Laboratory of Plant Molecular Physiology, Institute of Botany, Chinese Academy of Sciences, Beijing, China; 3 State Key Laboratory of Plant Physiology and Biochemistry, College of Biological Sciences, China Agricultural University, Beijing, China; National Institute of Plant Genome Research, India

## Abstract

Functional genomic elements, including transposable elements, small RNAs and non-coding RNAs, are involved in regulation of gene expression in response to plant stress. To identify genomic elements that regulate dehydration and alkaline tolerance in *Boea hygrometrica*, a resurrection plant that inhabits drought and alkaline Karst areas, a genomic DNA library from *B. hygrometrica* was constructed and subsequently transformed into *Arabidopsis* using binary bacterial artificial chromosome (BIBAC) vectors. Transgenic lines were screened under osmotic and alkaline conditions, leading to the identification of Clone L1-4 that conferred osmotic and alkaline tolerance. Sequence analyses revealed that L1-4 contained a 49-kb retroelement fragment from *B. hygrometrica*, of which only a truncated sequence was present in L1-4 transgenic Arabidopsis plants. Additional subcloning revealed that activity resided in a 2-kb sequence, designated *Osmotic and Alkaline Resistance 1* (*OAR1*). In addition, transgenic *Arabidopsis* lines carrying an *OAR1*-homologue also showed similar stress tolerance phenotypes. Physiological and molecular analyses demonstrated that *OAR1*-transgenic plants exhibited improved photochemical efficiency and membrane integrity and biomarker gene expression under both osmotic and alkaline stresses. Short transcripts that originated from *OAR1* were increased under stress conditions in both *B. hygrometrica* and *Arabidopsis* carrying *OAR1*. The relative copy number of *OAR1* was stable in transgenic *Arabidopsis* under stress but increased in *B. hygrometrica*. Taken together, our results indicated a potential role of *OAR1* element in plant tolerance to osmotic and alkaline stresses, and verified the feasibility of the BIBAC transformation technique to identify functional genomic elements from physiological model species.

## Introduction

Drought, alkaline and high calcium are major environmental factors in South-west China Karst landforms that limit plant growth and crop productivity [1], [2]. Improving crop tolerance to environmental stresses is thus beneficial for both the agriculture and ecosystem dynamics in the region. Drought stress has been intensively studied in plants, resulting in the identification of a large number of genes that play a potential role in tolerance mechanisms [3]. In contrast, tolerance to alkaline and high calcium stress has not been intensively studied in plants.

Many plant species in the Gesneriaceae family such as Boea hygrometrica, Haberlea rhodopensis, Ramando myconi, Metapetrocosmea peltata, Chirita heterotricha, Oreocharis flavida, and Paraboea rufescens [4] are well adapted to the Karst region, and grow in shady limestone crevices where the soil is alkaline [5], [6]. B. hygrometrica, H. rhodopensis and R. myconi, are also known as resurrection plants, a category of plants that are able to tolerate full desiccation (leaf relative water content <10%) and are viable after rehydration within 48 h [7]–[9].

Previously, it was reported that the thylakoid pigment-protein complexes and pigment contents were highly stable during desiccation and rehydration in *B. hygrometrica*, but were irreversibly lost in the desiccated leaves of a non-resurrection Gesneriaceae species *Chirita heterotrichia* [7]. The stabilization of photosynthetic apparatus during water deficit had also been reported on resurrection plants in *Haberlea spp.* and *Ramando spp.* [8], [9], indicating that these plants may have evolved distinct adaptive mechanisms to cope with desiccation. Plant responses to water deficit are complex, and these responses can be synergistically or antagonistically modified by the superimposition of other stresses [13]. It is unknown whether the concurrent environment factors such as high calcium and alkali evoke common adaptive mechanisms that dehydration triggers in these Gesneriaceae resurrection plants.

Genome-level regulation such as chromatin modification and assembly, transposable elements, small RNAs, and non-coding RNAs are involved in plant stress responses [14], [15].With the exception of *CDT-1* from *Craterostigma plantagineum* [16]–[18], genomic elements that regulate stress tolerance in resurrection plants have not been identified, which is largely due to the lack of genome sequence data and genetic analysis tools.

Binary bacterial artificial chromosome (BIBAC) vectors were developed for transformation of large genomic DNA fragments into plants. This technology can be used overcome the technical limitations in species where genetic transformation and genome sequences are not available [19]–[21]. Therefore it became a useful tool for phenotype-based screening of genomic elements. For example screening of a BIBAC library from *Thellungiella halophila* led to the identification of a clone with a 120–130-kb insert that is associated with improved salt tolerance [22]. Screening of a BIBAC library from *Leavenworthia alabamica* also led to the identification of 84 20-kb genomic clones with phenotypic effects such as short fruit and aborted seeds [23]. In this study, a BIBAC library was constructed with *B. hygrometrica* genomic DNA and used for generation of transgenic populations. A BIBAC clone that conferred osmotic or alkaline tolerance was identified, and the resident functional element that might be responsible for the improved osmotic and alkaline tolerance was assigned.

## Materials and Methods

### BIBAC library construction

*B. hygrometrica* plants were collected from a self-bred population grown in green house conditions in our lab. Therefore no specific permission was required for these collections. This study did not involve endangered or protected species. Leaves of young *B. hygrometrica* plants were ground in liquid nitrogen. The isolation of the nuclear DNA was conducted as described by Zhang et al. [24]. Nuclear DNA in the plugs was partially digested by *Bam*HI and analyzed by pulsed-field gel electrophoresis (PFGE) using a WD-2010 apparatus (Beijing Liuyi Instrument Factory, China) on 1% agarose gels in 0.5×TBE buffer at a 5-s pulse time of 6 V/cm, at 15°C for 15 h. Restriction fragments in a range from 40 to 90-kb were collected and ligated with the vector pCLD04541 [25], which was completely digested and dephosphorylated. Ligated DNA was transformed into *E. coli* strain DH10B electrocompetent cells (Gibco-BRL, USA) by electroporation using a Cell Porator and Voltage Booster System (Gibco-BRL, USA) as described by Zhang et al [26]. About 4,600 clones were obtained from selection media, and were arrayed in 12×384-well microtiter plates and maintained in −80°C.

### Analysis of BIBAC clones

Random clones from the BIBAC library were grown overnight at 37°C in LB medium containing 15 mg/L tetracycline (Amresco, USA). Plasmid DNA was isolated with the alkaline lysis method. Insert fragments were released from the pCLD04541 vector by digestion with *Not*I (TaKaRa, Japan) and subjected to PFGE performed as described above. The insert sizes of these clones were estimated using a lambda DNA ladder as the molecular-weight standard.

### Estimation of nuclear genome size

The absolute amount of nuclear DNA (i.e. genome size) in *B. hygrometrica* was estimated using flow cytometry analysis. The experimental material consisted of leaves of *B. hygrometrica*, with *Arabidopsis thaliana* serving as an internal reference standard. The histogram of relative DNA content was obtained after flow cytometric analysis of nuclei of *B. hygrometrica* and *Arabidopsis*, which were isolated, stained, and analyzed simultaneously. *B. hygrometrica* 2C DNA content = (*Boea* G1 peak mean)/(*Arabidopsis* G1 peak mean)×*Arabidopsis* 2C DNA content (*Arabidopsis* 1C = 125 Mb) [27].

### Transformation of BIBAC clones into *Agrobacterium* and *Arabidopsis*

The BIBAC clone was sequenced to obtain terminal sequences that were used to design primers for clone*-*specific designation. Plasmid DNA of BIBAC clones was isolated with the alkaline lysis method and transformed into *A. tumefaciens* strain GV3101 via electroporation. The electroporated GV3101 clones were selected on YEB medium containing 50 mg/L rifampincin, 50 mg/L kanamycin, and 35 mg/L gentamycin. Random colonies were incubated in liquid YEB medium with antibiotics (as above) for 2 days at 28°C with shaking at 170 rpm and confirmed by PCR using primer pairs designed according to the appropriate clone*-*specific terminal sequences. *Agrobacterium* clones transformed with L1*-*4 were incubated in liquid YEB medium with antibiotics (as above) at 28°C, with shaking at 170 rpm. When the OD_600_ value of the culture increased to about 0.8, 500 µl of the culture was transferred to a 50 ml culture for continued growth. These cultures were subcultured 4 times, for approximately 10 hours for each passage. 1 µL of the first culture and 1 µL of the fifth culture were used to analyze the integrity of the BIBAC DNA in *Agrobacterium* using the multiple*-*marker PCR*-*based method [20].

Floral*-*dip transformation of *Arabidopsis* was conducted using the Columbia ecotype [28]. Transgenic plants were confirmed by PCR amplification using the BIBAC terminal specific primers. Homozygous lines were selected through two further rounds of selection on plates containing 50 mg/L kanamycin. Kanamycin*-*resistant T_1_ plants were transferred to soil and seeds were collected. These T_2_ seeds were sown on plates containing 50 mg/L kanamycin. The ratio of green to yellow seedlings of each line was analyzed with a Chi*-*square test. The lines with a 3∶1 ratio of survival on kanamycin were selected and grown to maturity. T_3_ seeds were collected and sown again on plates containing 50 mg/L kanamycin, and lines with 100% survival were considered as homozygous. The relative copy numbers and expression levels of the transgenes were checked by quantitative real-time PCR using genomic DNA and reversed transcribed DNA from RNA from the transgenic plants as templates, respectively. Only T_3_ seeds of homozygous lines were used for further experiments.

Thermal asymmetric interlaced PCR (Tail*-*PCR) [29] was used to determine the insertion site of L1*-*4 in transgenic line L1*-*4*-*2. Genomic DNA was extracted using the CTAB method [30], and used as the template for Tail-PCR. Three primers, SP1, SP2, and SP3 were designed according to the adjacent sequences of the multiple cloning site of pCLD04541, using AD1 as the degenerate primer (Table S1 in File S1.).

### Examination of stress tolerance in transgenic lines

T_3_ seeds of transgenic lines were surface sterilized and placed on 1/2 Murashige & Skoog (MS) agar plates, and then cultivated vertically at 22°C for germination with a 16 h light/8 h dark cycle. 3 day-old seedlings were transferred to 1/2 MS agar plates that were saturated overnight with different concentrations of PEG 8000 (25% and 40% (w/v) PEG) for osmotic screening [31]; to 1/2 MS agar plates which were adjusted with potassium hydroxide to pH 5.6, 8.5, or 9.0 respectively, for alkaline screening; and to agar plates containing 60 mM or 80 mM CaCl_2_ for high-calcium stress screening. The location of the seed lots were kept consistent in each treatment in one set of experiments but arranged randomly in different sets of repetitions. For each treatment, at least three independent experiments were conducted with at least three plates with 6 seedlings per line per plate was assayed. Photographs were taken, total root length and physiological parameters were determined after 2 weeks of growth. The total length of primary root and lateral roots were measured with ImageJ software. The empty vector control (pCLD04541) transformed plants showed no difference compared to the wild-type (Col-0) on PEG and alkali stress condition (Figure S1 in File S1.), therefore were not included in the subsequent phenotyping test. For soil dry treatment, T_3_ seeds of transgenic lines were germinated on 1/2 MS agar plates and 5 day-old seedlings were transferred to soil in pots for dehydration for 7 days. Three replicates of 25 seedlings were tested in each treatment. In all cases, wild-type seed batches that were generated at the same time as the transgenic seed lines were germinated and transferred in parallel with the transgenic plants as controls.

### Physiological parameter determination

Leaf relative water content (RWC) was estimated according the following formula: (RWC, %) = (fresh weight − dry weight)/(turgid weight − dry weight)×100. Photochemical efficiency (Fv/Fm), and the extent of electrolyte leakage were measured as described previously [10]. Leaves were fixed with absolute ethyl alcohol for 2–3 min and used for stomata observation with light microscopy (B204LED, China). The experiments were performed twice, with three independent leaves for each treatment at each time point.

### Shotgun sequencing

The BAC plasmid of L1-4 was purified using the QIAGEN Large-Construct Kit. Ultrasonically-broken and sheared BAC DNA (1.5–3-kb) was ligated into the pUC19 vector and transformed into *E. coli* strain Top10. The generated shotgun subclones were then sequenced from both ends using the dideoxy chain termination method using BigDye Terminator Cycle Sequencing V3.1 Ready Reaction (Applied Biosystems) on ABI3730xl Capillary Sequencing machines (Applied Biosystems). The subclones were sequenced to generate 8–10 fold coverage. The Phred-Phrap program (University of Washington, Seattle, WA, USA; http://www.phrap.org/phredphrapconsed.html) was used to assemble the shotgun sequences and gap-closing of each BAC [32], [33].

### Subclone library construction

The plasmid DNA of BIBAC L1-4 was partially digested by *Sau*3A. Fragments between the sizes of 5 and 8-kb were collected and ligated into the pCLD04541 plasmid which was completely digested by *Bam*HI and dephosphorylated. The recombinant DNA was transformed into *E. coli* EPI300 competent cells by electroporation. S3, S21, S32, and S35 were identified by PCR with different pairs of primers: 1 kF and 1 kR, 3 kF and 3 kR, 4 kF and 4 kR, 5 kF and 5 KR, 14 kF and 14 kR, 19 kF and 19 kR, 44 kF and 44 kR, 46 kF and 46 kR, and 47 kF and M13R-48, respectively (Table S1 in File S1.).

### Sequence analysis

The open reading frames (ORFs) in the genomic sequences were predicted with the gene-finding tools FGENESH (http://linux1.softberry.com) using the *Arabidopsis* dataset with default settings. Repetitive elements were searched with the program RepeatMasker (http://www.repeatmasker.org/cgi-bin/WEBRepeatMasker). The retrotransposons were identified with LTR_ Finder (http://tlife.fudan.edu.cn/ltr_finder/). The gene identities were predicted with BLAST analysis (http://blast.ncbi.nlm.nih.gov).

### RNA isolation and Real-time PCR

Total RNA was isolated using the TRIzol method with RNAiso Plus (Takara, D9108B). After digestion with DNase I, it was reverse transcribed into cDNA with Oligo(dT)18 primer using M-MLV reverse transcriptase, and used as template for PCR amplification. *ACTIN2* and *18S* were used as internal references for transgenic lines and *B. hygrometrica* gene expression determination, respectively. Real-time PCRs were performed in a Mastercycler ep realplex apparatus (Eppendorf, Hamburg, Germany) with SYBR Green Realtime PCR Master Mix (TOYOBO, Japan). Specific primers were listed in Table S1 in File S1.

### Determination of *OAR1* relative copy number

Relative copy number of *OAR1* was assessed by quantitative real-time PCR using OAR1-1F and OAR1-1R primers and genomic DNA from *B. hygrometrica* and transgenic *Arabidopsis* lines S21-3 and S21-14. *NPTII* gene in the transgene cassette was used as an internal control for determination of the relative copy number of *OAR1* in transgenic *Arabidopsis* lines. *NPTII* gene in the plasmid DNA of the empty vector pCLD04541 was used as an external control for determination of the relative copy number of *OAR1* in the *B. hygrometrica* genome.

### Accession numbers

The *OAR1* nucleotide sequence has been submitted to NCBI with accession number KF425673.

## Results

### Construction and characterization of a *B. hygrometrica* BIBAC library

**Figure 1:**
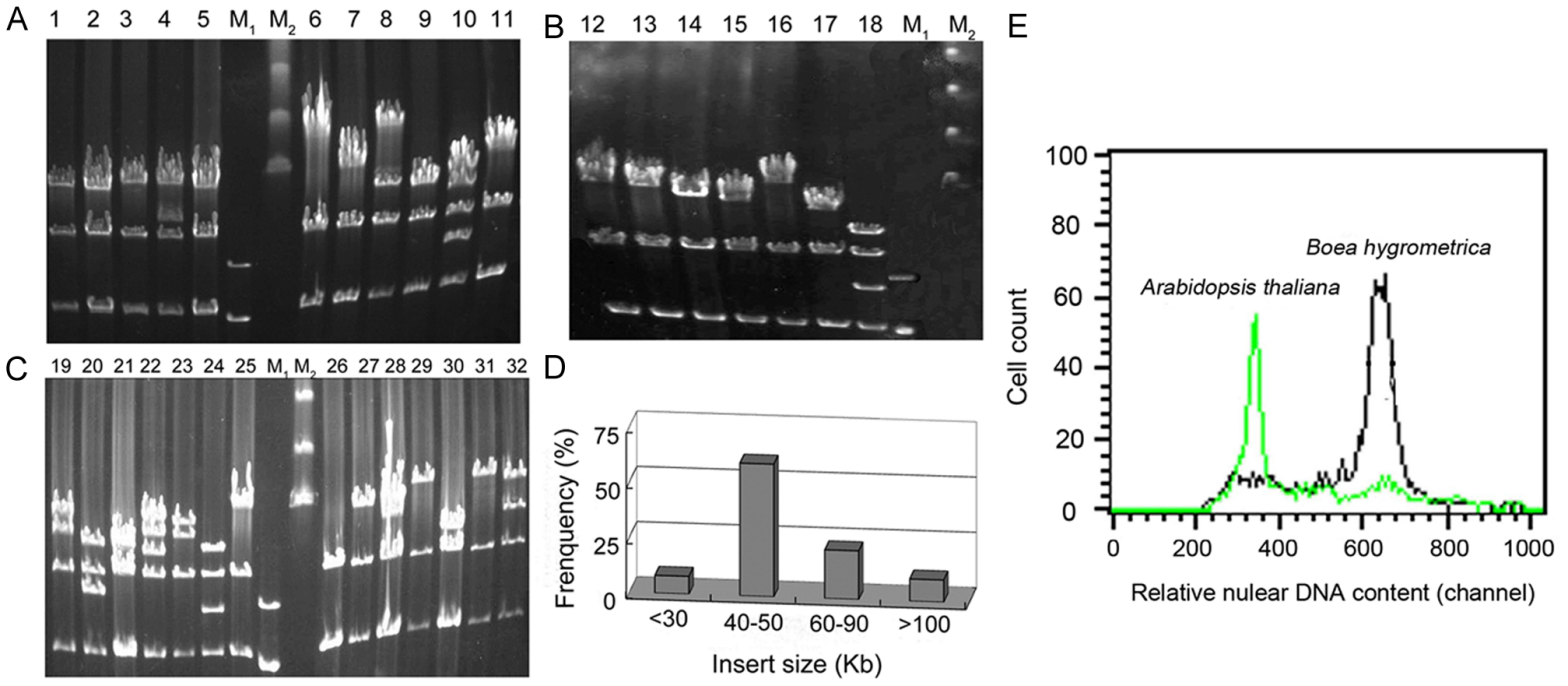
Analysis of insert size for the *Boea hygrometrica* BIBAC library and determination of genome size of *B. hygrometrica* by flow cytometry. (A–C) Pulsed*-*field gel electrophoresis (PFGE) patterns of 32 representatives of the 50 random BIBAC clones that were digested with *Not*I. PFGE gels were stained with ethidium bromide. M_1_, marker with bands of size 3, 5 and 8-kb; M_2_, Lamda ladder PFG marker with band sizes of 48.5, 97, 145 and 194-kb. (D) Insert size distributions of the 50 clones randomly selected from the *B. hygrometrica* BIBAC library. (E) Determination of genome size of *B. hygrometrica* by flow cytometry. *Arabidopsis* served as internal reference standard. *B. hygrometrica* 2C DNA content = (*Boea* G1 peak mean)/(*Arabidopsis* G1 peak mean) ×*Arabidopsis* 2C DNA content, the ratio of G1 peak means (*B. hygrometrica*: *Arabidopsis*) was 1.86, hence the 2C DNA amount of *B. hygrometrica* was estimated as 480 Mb (*Arabidopsis* 1C = 125 Mb).

A BIBAC genomic library was constructed for *B. hygrometrica* from nuclear DNA, containing about 4,600 clones in total. To estimate the quality of this library, the insert sizes of 50 randomly selected clones from the library were analyzed by digesting the plasmid DNA with *Not*I and subsequent separation by pulsed-field gel electrophoresis (PFGE). The results are presented in Figure 1 (A–D). The majority of these clones contained inserts with sizes ranging from 40 to 90-kb. Because 10% of the clones have inserts larger than 100-kb, the average insert size was 62-kb. Among these 50 clones, one did not contain an insert, accounting for 2% of the clones analyzed. Thus the frequency of clones lacking an insertion was lower than that of the BIBAC libraries made for tomato [34], petunia [35], rice [36], *Arabidopsis thaliana* ecotype Landsberg [37], or chickpea [38], which were 10%, 6.5%, 4.8%, 17.6%, and <5%, respectively. The low frequency of empty constructs and the fact that each BAC clone contained different insertion sizes implies that the *B. hygrometrica* BIBAC library was of good quality.

To determine the genome coverage of this library, the nuclear DNA content of *B. hygrometrica* was estimated by flow cytometry. Using *Arabidopsis* as an internal reference, the haploid genome size of *B. hygrometrica* was calculated to be 240 Mb (Figure 1E). Based on the average insert size of 62-kb the coverage of the library is approximately 1.18 haploid genome equivalents.

### Construction of populations of transgenic *Arabidopsis* lines carrying *B. hygrometrica* BIBAC clones

288 BIBAC clones were subjected to BAC terminal sequencing to obtain sequence information for designing primers that could be used to generate markers of each clone. Among these, 172 unique BIBAC plasmids were selected for transformation into *Agrobacterium* and subsequently into *Arabidopsis*, as indicated by distinct PCR products using the clone-specific primers. In total, 43 BIBAC clones were successfully transformed into *Arabidposis* and 213 transgenic lines were generated. For 37 of these lines, >3 independent transformants were obtained and subsequently used for phenotypic analyses.

### BIBAC clone L1-4 conferred osmotic and alkaline tolerance in transgenic *Arabidopsis*

**Figure 2:**
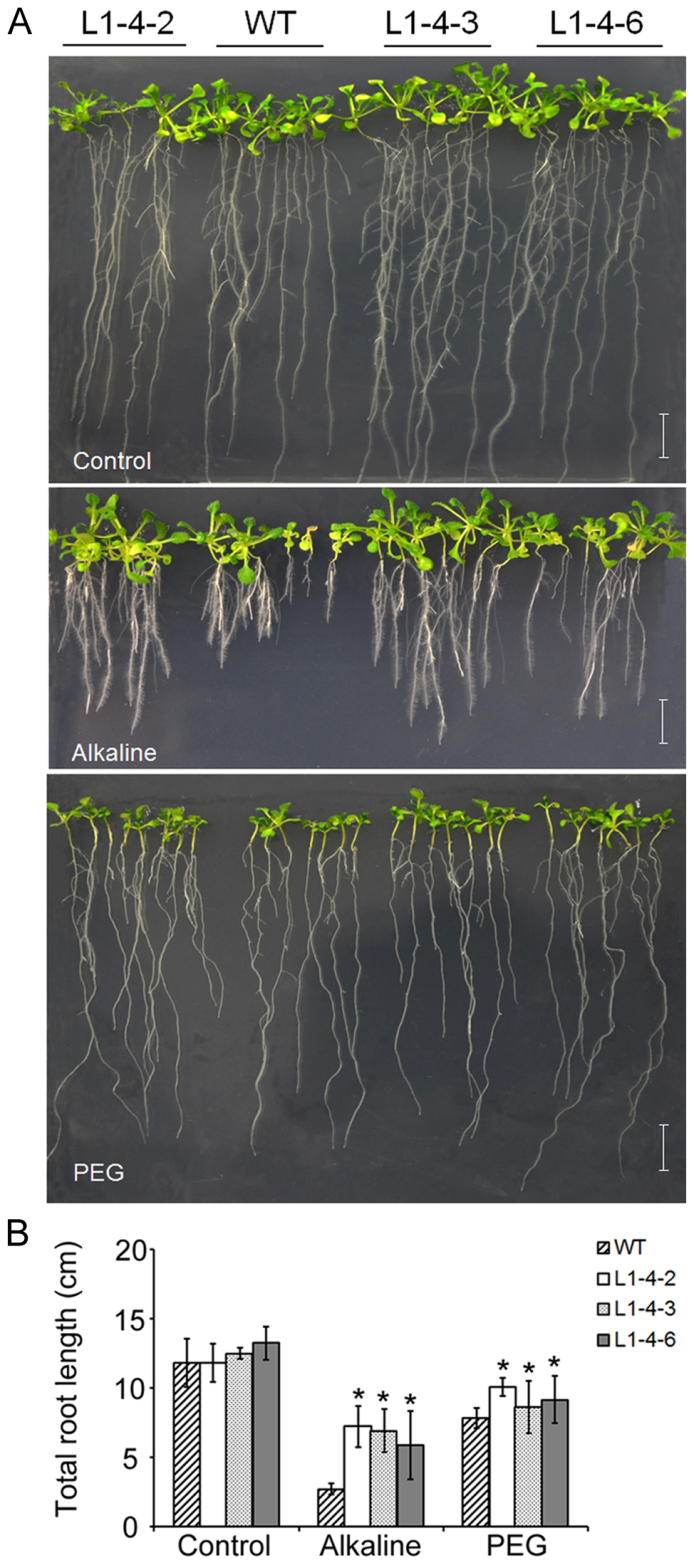
Phenotypes of L1-4 transgenic plants under alkaline and osmotic stress. (A) Seedlings grown on agar plates containing 1/2 MS with pH5.6 (control), pH8.5 and soaked with 25% PEG 8000. (B) Total root length of transgenic and wild-type plants grown on alkaline media (pH 8.5) and osmotic stress with 25% PEG 8000 for 14 d. 3 day-old seedlings were transferred to control media or media with alkaline pH or 25% PEG 8000 media. Wild-type seed batches that were generated at the same time as the transgenic seed lines were germinated and transferred in parallel with the transgenic plants as controls. n = 18, Bar = 1cm, data are shown as means ± SD.

To identify genomic DNA fragments of *B. hygrometrica* that are able to improve osmotic, alkali, and/or high calcium tolerance, T_3_ generation transgenic plants were subjected to screening under osmotic, alkaline, or high calcium stress conditions. The results revealed that transgenic lines carrying the BIBAC clone L1-4 exhibited tolerance to both osmotic and alkali stresses. These transgenic plants grew well on 1/2 MS plates (pH 5.6), showing no obvious difference from the wild-type. However, transgenic plants exhibited better growth as shown by the bigger rosette, longer total roots (primary and more lateral roots together) on alkaline media (pH 8.5) or media with 25% PEG, as compared to the wild-type and “empty vector” control plants (Figure 2, Figure S1 in File S1.). The alkali resistant phenotype was further confirmed by the higher ratio of plant survival in transgenic lines on alkaline media (pH 9.0) than that in wild-type (Figure S2 in File S1.). On PEG-mediated osmotic stress conditions, the transgenic plants exhibited thicker and longer lateral roots compared to the wild type (Figure 2). The osmotic resistant phenotype was further supported by the higher survival rates of the transgenic plants of L1-4 than the wild-type under soil drought conditions (Figure S3 in File S1). However, these transgenic lines did not show tolerance to high calcium (data not shown). These data suggested that a functional fragment of genomic DNA carried by BIBAC clone L1-4 was able to confer osmotic and alkaline tolerance in *Arabidopsis*.

To check if the observed phenotypes of transgenic plants resulted from an insertional mutagenesis event, the insertion site of L1-4 in transgenic line L1-4-2, which exhibited the strongest osmotic and alkaline tolerance among the transgenic lines, was examined. The result indicated that L1-4 DNA inserted in the interval of a non-coding region between two alpha tubulin (TUA) genes in *Arabidopsis* chromosome 5,897 bp up-stream of *TUA3* (reverse orientation) and 1703 bp up-stream of *TUA5* (forward orientation) on chromosome 5 (Figure S4A in File S1). Semi-quantitative RT-PCR revealed that *TUA3* and *TUA5* expression was reduced in L1-4 transgenic plants (Figure S4B in File S1).

### L1-4 contained a cluster of nested LTR-retrotransposons

The insert size of BIBAC L1-4, as determined by a complete *Not*I digestion and PFGE, was approximately 50-kb (Figure S5 in File S1). To identify the specific genetic element that is responsible for the osmotic and alkaline pH-resistant phenotypes, shotgun sequencing was performed to obtain the full sequence information of the insert DNA in L1-4. Sequence assembly resulted in a continuous 49,387 bp contig with the GC content of 45.49%. Using the sequence to query the GenBank nucleotide database, we detected several discontinuous DNA fragments with similarities to the genomic sequences of *Mimulus guttatus*, grape, tomato, populous, and pineapple, with the highest similarity of 70% and query coverage of up to 17%. Besides, TBLASTX analysis did not detect any similarity to known proteins with the exception of gag-pol polyproteins [39], which are generally components of retrotransposons (retroelements).

**Figure 3:**
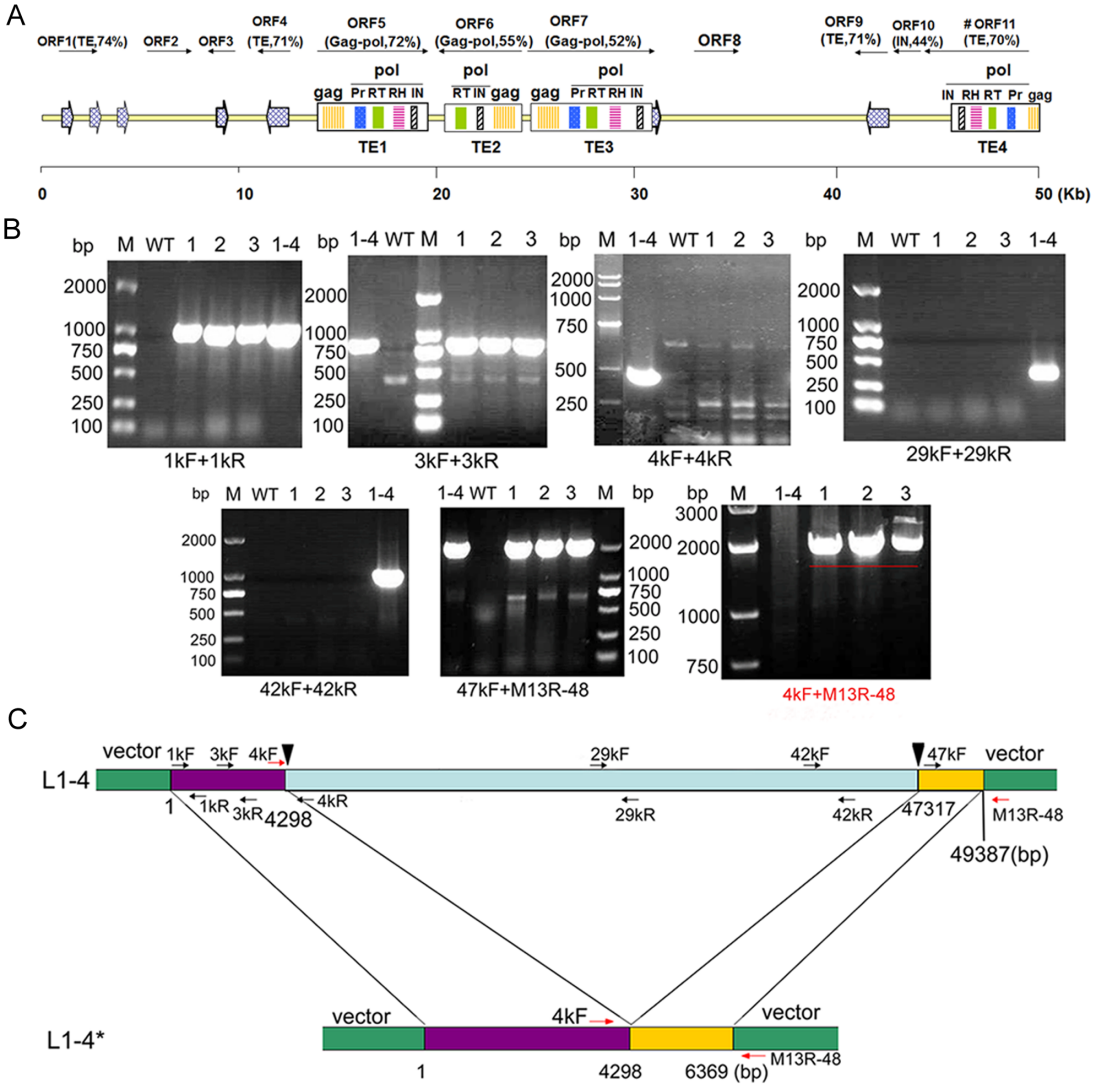
Annotation of insert sequence in BIBAC clone L1*-*4 and truncation of L1-4 in the transgenic plants. (A) ORFs predicted with the program FGENESH were annotated by TBLASTX, transcriptional orientation is indicated with an arrow. A schematic diagram was drawn to show the position of the predicted LTRs (grid arrows) and retrotransposons. The intact LTR*-*retrotransposon identified by LTR*-*finder is indicated in bold. gag, capsid-like protein; pol, polpolyprotein; IN, integrase; PR, pepsin-like aspartate proteases; RH, RNase H; RT, Reverse transcriptase; LTR, long terminal repeat; TE, transposable element. (B) Assay of PCR amplification using specific primers corresponding to different regions in L1-4. Primers were designed to amplify fragments located at 1, 3, 4, 14, 29, 42, and 47–49-kb of L1-4, as indicated on the bottom of each picture and listed in (**C**) and Table S1 in File S1. M, DNA marker with the size of 100, 250, 500, 750, 1000 and 2000 bp; 1–4, plasmid DNA of BIBAC clone L1*-*4; WT, genomic DNA of *Arabidopsis* wild*-*type Col*-*0; 1, 2, 3, genomic DNA of three independent transgenic lines of L1*-*4. The PCR fragments were separated on 1% agarose gels. (**C**) Schematic diagram of L1*-*4 and L1*-*4*. The positions and orientation of primers are indicated by arrows.

To identify possible protein-coding sequences, the FGENESH algorithm was used for ORF prediction in the L1-4 insert. This resulted in the identification of 11 ORFs, 10 of which were complete (Figure 3A). Standard BLASTP analysis failed to find any homologues for ORF2, 3, or 8; but it revealed homologues to long terminal repeats (LTRs) for ORF1, 4, and 9, as well as gag-pol polyproteins of LTR-retrotransposons for ORF5, 6, 7, and 11, suggesting that L1-4 may indeed consist of LTR-retrotransposons, which is a major type of transposons (transposable elements, TEs) in higher plants [40]. In total, 3 gypsy (TE1, TE3 and TE4), and 1 copia (TE2) were predicted in the L1-4 contig according to the organization of the *gag* and *pol* genes. LTR finder and RepeatMasker were then used to predict the corresponding LTR of the identified retrotransposons. Only one pair of LTRs was identified as clamped LTRs of TE1, while the others were solo-LTR or incomplete. TE1 and TE4 were highly homologous (>95%) in different orientations. TE2 and TE3 were located inside the LTR pair*-*clamped region downstream of the 3′ terminus of TE1 (Figure 3A). Thus the whole contig of L1-4 was constituted of four nested and truncated retrotransposons. Such genomic structure has been reported previously in both plants and animals [41]–[43]. The middle sections of both TE1 and TE4 show a remarkable resemblance to *Arabidopsis* LTR*-*retrotransposons *AtGP1* and *AtGP2* [44]. Besides, 130 dimer*-* and 42 trimer*-*microsatellites were detected in the 49-kb sequence. These data showed that L1*-*4 contained a region of the *B. hygrometrica* genome with short tandem repeated transposons and nested transposons.

### L1-4 became truncated in the transgenic *Agrobacterium* and *Arabidopsis*

Large DNA fragments are unstable after transformation into *Agrobacterium* [45]. The existence of multiple transposon*-*coding sequences in L1*-*4 raised the possibility of DNA deletion, re*-*insertion, and rearrangement. To check if the transgenic plants carried an intact fragment of the L1*-*4 BIBAC clone, primers were designed according to the shotgun sequence to amplify individual regions of the L1*-*4 sequence. As shown in Figure 3B, correct PCR products were obtained with all tested primer pairs when L1*-*4 plasmid DNA was used as template. However, when genomic DNA from three independent L1*-*4 transgenic lines were used as templates, correct PCR products were produced only with the primers within 0–4 and 47–49-kb of L1*-*4 insert. This suggested that only two terminal sequences of L1*-*4 had been integrated into the *Arabidopsis* genome in the transgenic lines. The loss of the middle part of the L1*-*4 sequence in transgenic plants was further confirmed by sequencing of the amplified products, which showed continuous sequence homology from 4,096 to 4,298 bp and from 47,317 to 48,145 bp in the L1*-*4 contig. Thus it was evident that the transgenic lines harbor only an internally truncated L1*-*4 sequence, which was 6,369 bp in length, as shown in Figure 3C. To distinguish it from the intact plasmid L1*-*4 sequence, the truncated 6,369 bp fragment was designated as L1*-*4*.

Because all three independent transgenic lines exhibited the same deletion, we speculated that the deletion had occurred in *Agrobacterium*. To address this possibility, the insert DNA in the L1*-*4 transformed *Agrobacterium* strain stored in *−*80°C for different periods was analyzed. The PCR amplification of different regions of L1*-*4 indicated that the inserted DNA was intact in *Agrobacterium* cultured directly from the stock in *−*80°C for both six months and three years (Figure S6A–C in File S1). However, when *Agrobacterium* were continuously subcultured in liquid medium at 28°C, some regions of L1*-*4 were no longer detectable in some of the randomly selected clones (Figure S6D–E in File S1). These results indicated that the large insert DNA in the plasmid of *Agrobacterium* was stable when stored in −80°C for three years, but not during sequential liquid subculturing at 28°C. This is probably the reason for the truncation of L1-4 in transgenic *Arabidopsis*.

### Identification of the subclones containing partial sequences of L1-4*

**Figure 4:**
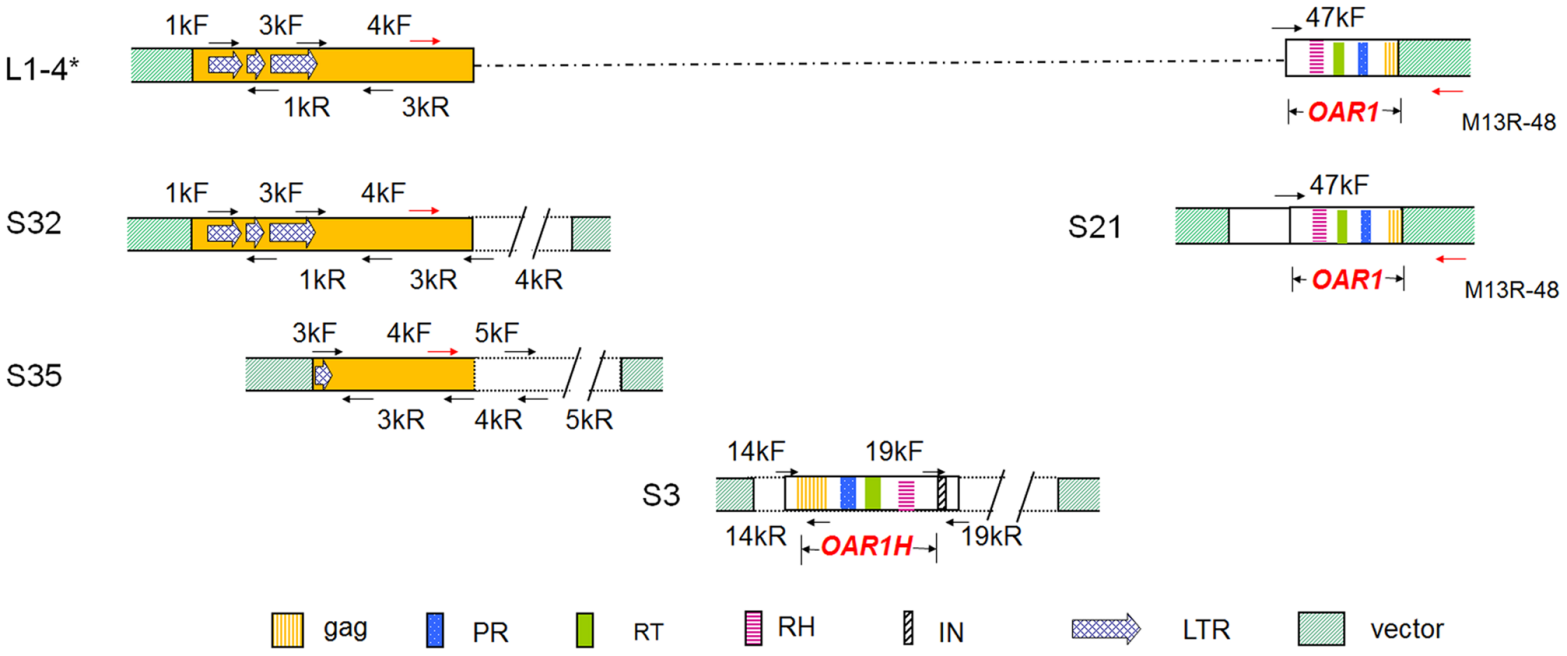
Schematic diagram of subclones containing partial sequences of L1-4*. Pairs of primers were designed according to sequence of L1-4, as indicated in Figure 3C. Subclone S21 contains 46–49-kb of L1-4 and 4–6-kb of L1-4*; subclones S32 and S35 contain 0–4-kb of L1-4 and L1-4*; subclone S3 contains 14–19-kb of L1-4. gag, capsid-like protein; pol, polpolyprotein; IN, integrase; PR, pepsin-like aspartate proteases; RH, RNase H; RT, Reverse transcriptase; LTR, long terminal repeat; TE, transposable element.

To dissect the particular genomic element that was responsible for the improved osmotic and alkaline tolerance of transgenic *Arabidopsis*, a subclone library was constructed containing 5–8 kb fragments of the L1-4 large insert DNA produced by partial digestion using *Sau*3A. The library consisted of 200 clones and covered >20 times of the L1-4 insert sequence. Subclones containing partial sequences of L1-4* were identified by PCR and confirmed by sequencing. The identified subclones S32 and S35 carried sequence corresponding to 1–4,298 bp of L1-4. Subclone S21 carried sequence corresponding to 46,317–49,387 bp of L1-4 (Figure 4). These constructs were transformed into *Arabidopsis*, and 20, 12, and 2 transgenic lines were obtained for the S21, S32, and S35, respectively.

### Identification of the genetic loci responsible for osmotic and alkaline tolerance

**Figure 5:**
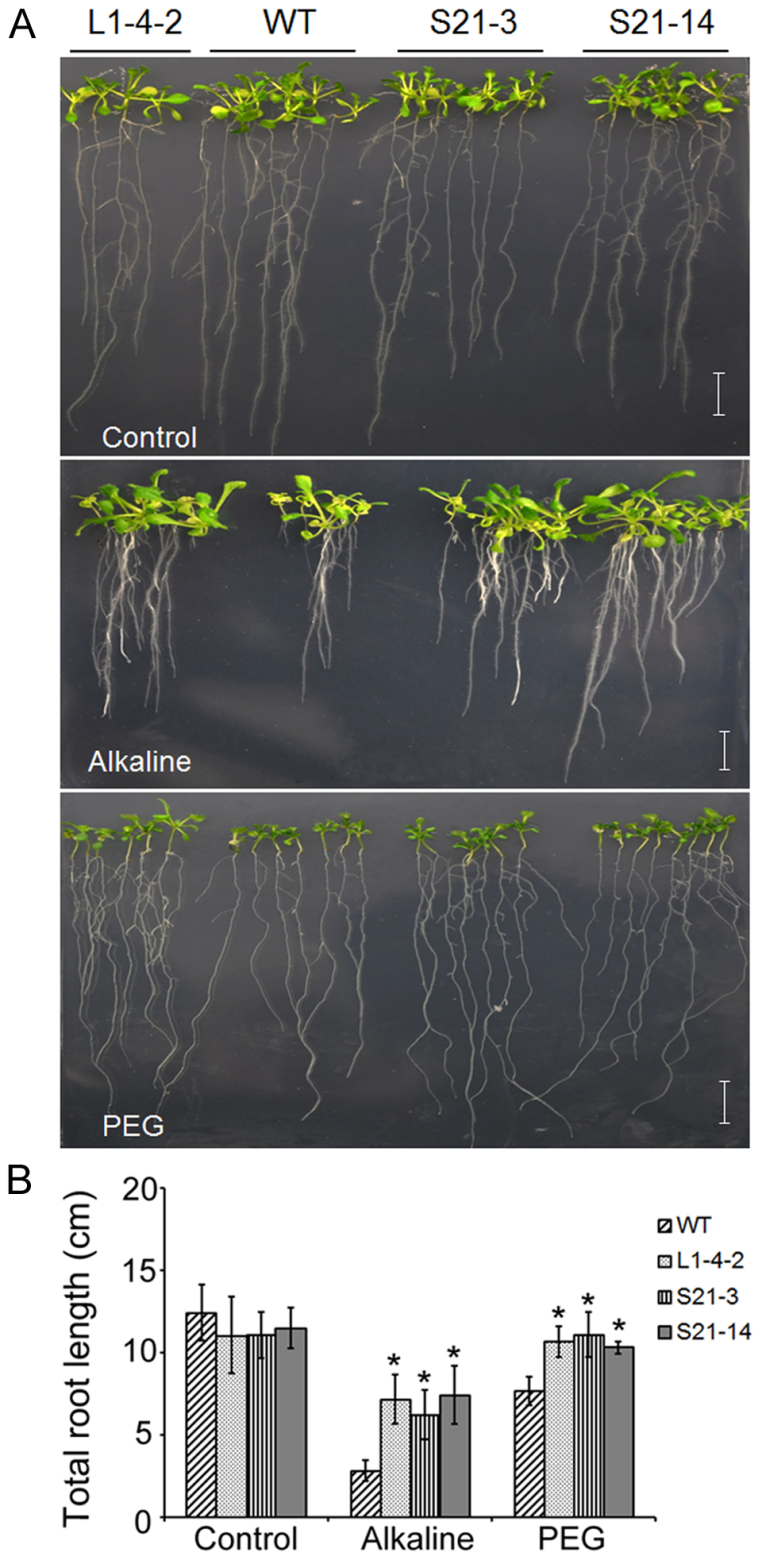
Phenotype comparison of the wild-type and transgenic plants harboring L1-4 and S21 under alkaline and osmotic stresses. (A) Seedlings grown on 1/2 MS agar plates adjusted to pH 5.6 and pH 8.5, and 1/2 MS agar plates saturated with 25% PEG 8000. (B) Total root length of plants on 1/2 MS agar plates adjusted to pH 5.6 and pH 8.5, and 25% PEG 8000 agar plates for 14 d.

Two transgenic lines from each construct of S21, S32, and S35 were assayed for osmotic and alkaline tolerance along with L1-4 transgenic and wild-type plants. Most of these transgenic lines grew similarly to the wild-type on 1/2 MS plates with or without PEG, or adjusted to pH 5.6 or 8.5. Only the plants harboring S21 displayed tolerance to alkaline and osmotic stresses, similar to the L1-4 transgenic plants, as indicated by the vigorous growth of shoots and improved root growth, including longer primary roots and more lateral roots, on the alkaline and PEG plates (Figure 5). The transgenic plants grow normally in soil, showing no obvious difference from the wild-type under unstressed conditions. However, when the seedlings were grown under soil drought condition in parallel with line L1-4-2 and the wild-type plants, the survival rates of S21 and L1-4 transgenic plants were significantly higher than the wild-type (Figure S3 in File S1), despite that the drought-resistant phenotype was observed only when the plants were young.

These observations indicated that the genetic element that conferred plant alkaline and drought tolerance in L1-4* was also located in S21 subclone. Alignment of the L1-4* and S21 sequences revealed that an overlapping sequence of 2076 bp, representing the 47,317–49,387 bp region of L1-4, hereby designated Osmotic and Alkaline Resistance 1 (*OAR1*). *OAR1* contains part of ORF11 of L1-4, which encodes a partial gag-pol transcript for RNase H, reverse transcriptase and protease (Figure 4).

**Figure 6:**
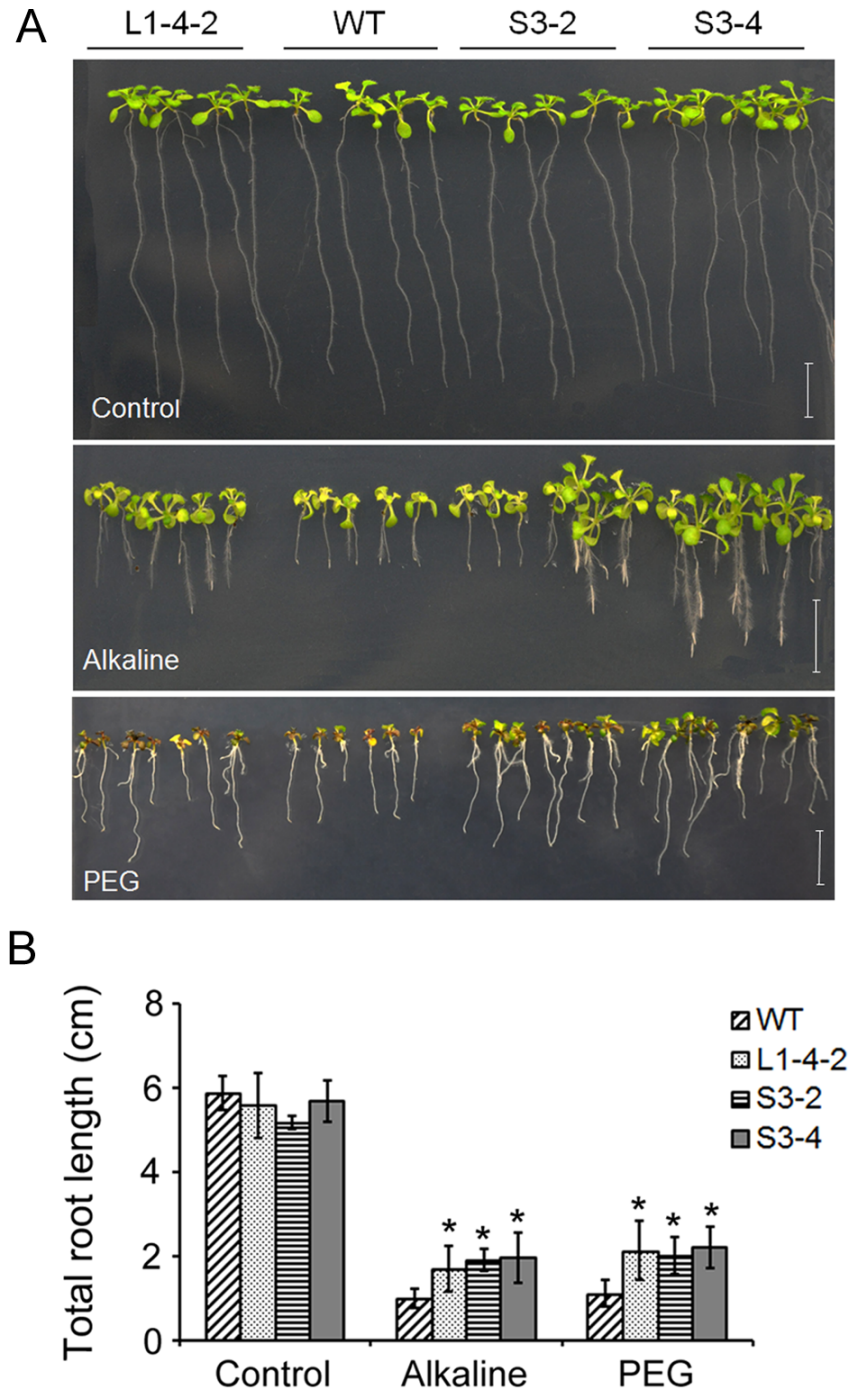
Phenotype comparison of the wild-type and transgenic plants harboring L1-4 and S3 under alkaline and osmotic stresses. (A) Seedlings grown on 1/2 MS agar plates adjusted to pH 5.6 (control) and pH 9.0, and 1/2 MS agar plates saturated with 40% PEG 8000. (B) Total root length of the wild-type and transgenic plants on 1/2 MS agar plates adjusted to pH 5.6 and pH 9.0, and on PEG 8000 agar plates for 10 d. n = 18, Bar = 1 cm. Data are shown as means ± SD.

Because *OAR1* is highly homologous to TE1 that is located in the 14–19-kb region of L1-4, subclone S3 containing TE1 (containing an *OAR1*-homologous sequence in the middle, *OAR1H*) was identified and transformed into *Arabidopsis* (Figure 4). Similar to the S21 transgenic plants, transgenic plants carrying S3 also displayed the improved tolerance to alkaline and osmotic stress treatments (Figure 6). Furthermore, over-expression of *OAR1* under the control of the *35S* promoter revealed a similar osmotic and alkaline stress tolerance phenotype (Figure S7 in File S1). In total, 4 independent transgenic lines of L1-4, 4 transgenic lines of S21, 5 transgenic lines of S3 and 3 lines of *35S*::*OAR1* displayed similar phenotypes, which suggested that the phenotype was not simply the result of positional effects. Thus, our results demonstrate that *OAR1* and *OAR1H* in L1-4 and its subclones are functional elements to confer plant tolerance to alkaline pH and drought stresses.

On the other hand, no difference was detected when the transgenic plants harboring S32 and S35 were compared to the wild-type under non-stressed or PEG/alkaline-stressed conditions (Figure S8 in File S1). As these plants were produced, propagated and phenotypic assayed in parallel with S21 transgenic plants, the failure of detecting visible PEG and alkaline resistant phenotypes in S32 and S35 transgenic plants not only enabled us to define the functional genetic element in L1-4* to *OAR1* that was common with L1-4* and S21, but also provided good negative controls to help to eliminate the possible effects of the antibiotic resistance marker on the PEG and alkaline resistant phenotypes of the L1-4 and S21 transgenic plants.

### Physiological characterization of the osmotic and alkaline tolerance in transgenic plants harboring *OAR1*

**Figure 7:**
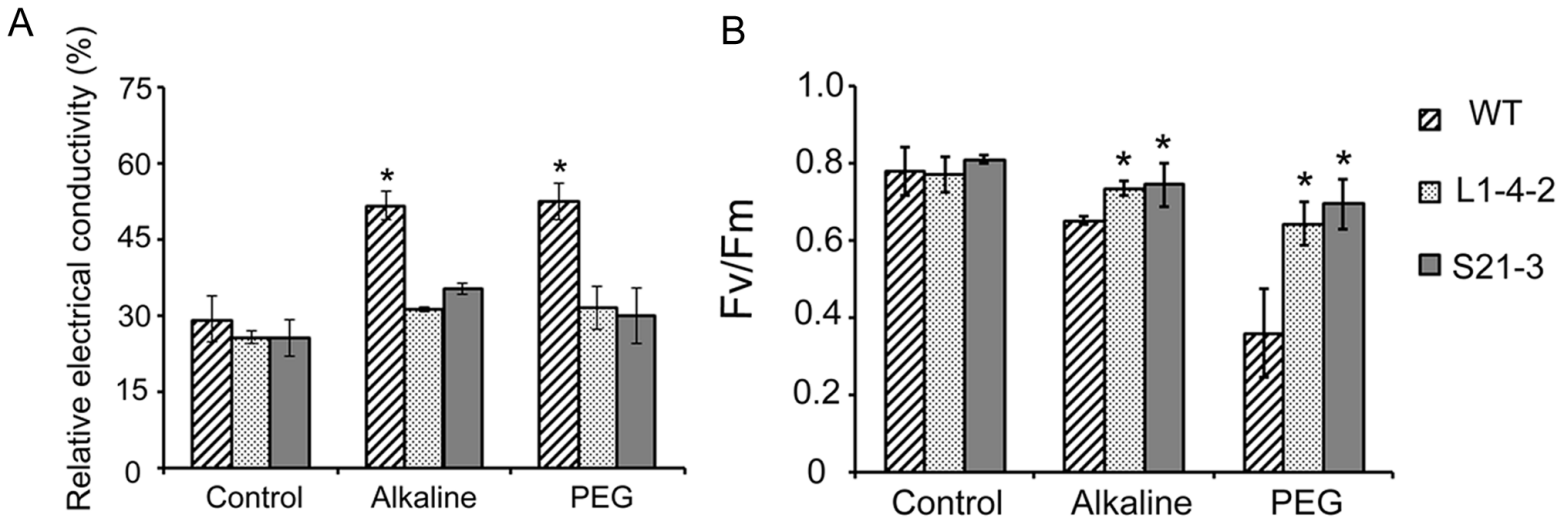
Physiological characterization of the L1-4 and S21 transgenic plants. (A) Electrical conductivity and chlorophyll fluorescence Fv/Fm characteristics (B) of seedlings grown on 1/2 MS (control) and alkaline (pH 9.0), or PEG plates (40% PEG 8000) following 2 weeks of growth. n = 18. Data are shown as means ± SD.

Transgenic plants harboring *OAR1* were further analyzed by the electrolyte leakage and photochemical efficiency using two representative lines that displayed the strongest osmotic and alkaline tolerance, L1-4-2 and S21-3, in parallel with the wild-type. The results revealed no difference in electrolyte leakage or photochemical efficiency (Fv/Fm) between the transgenic and wild-type plants grown under unstressed conditions. However, under alkaline or osmotic stresses, the electrolyte leakage increased to 51–53% and the Fv/Fm declined to 0.4 and 0.6 in wild-type plants under osmotic and alkaline stresses, while that of transgenic lines remained around 29–35% and 0.7, respectively (Figure 7). The difference between the wild-type and transgenic plants harboring *OAR1* was significant, indicating that the stress-triggered decline of cytomembrane integrity and photochemical efficiency was prevented in the transgenic plants harboring *OAR1*, which was consistent with the observed growth phenotype. Examination of the relative water content (RWC) and the stomata in the plants surviving on PEG plates showed no difference in these parameters between transgenic line S21-3 and the wild-type plants under unstressed and PEG-stressed conditions (Figure S9 in File S1). Thus, the survival of *OAR1*-containing transgenic plants on PEG plates might be due to the maintenance of membrane integrity and photochemical efficiency and not due to differences in rates of water loss.

### The expression and copy number of *OAR1* in transgenic *Arabidopsis*

**Figure 8:**
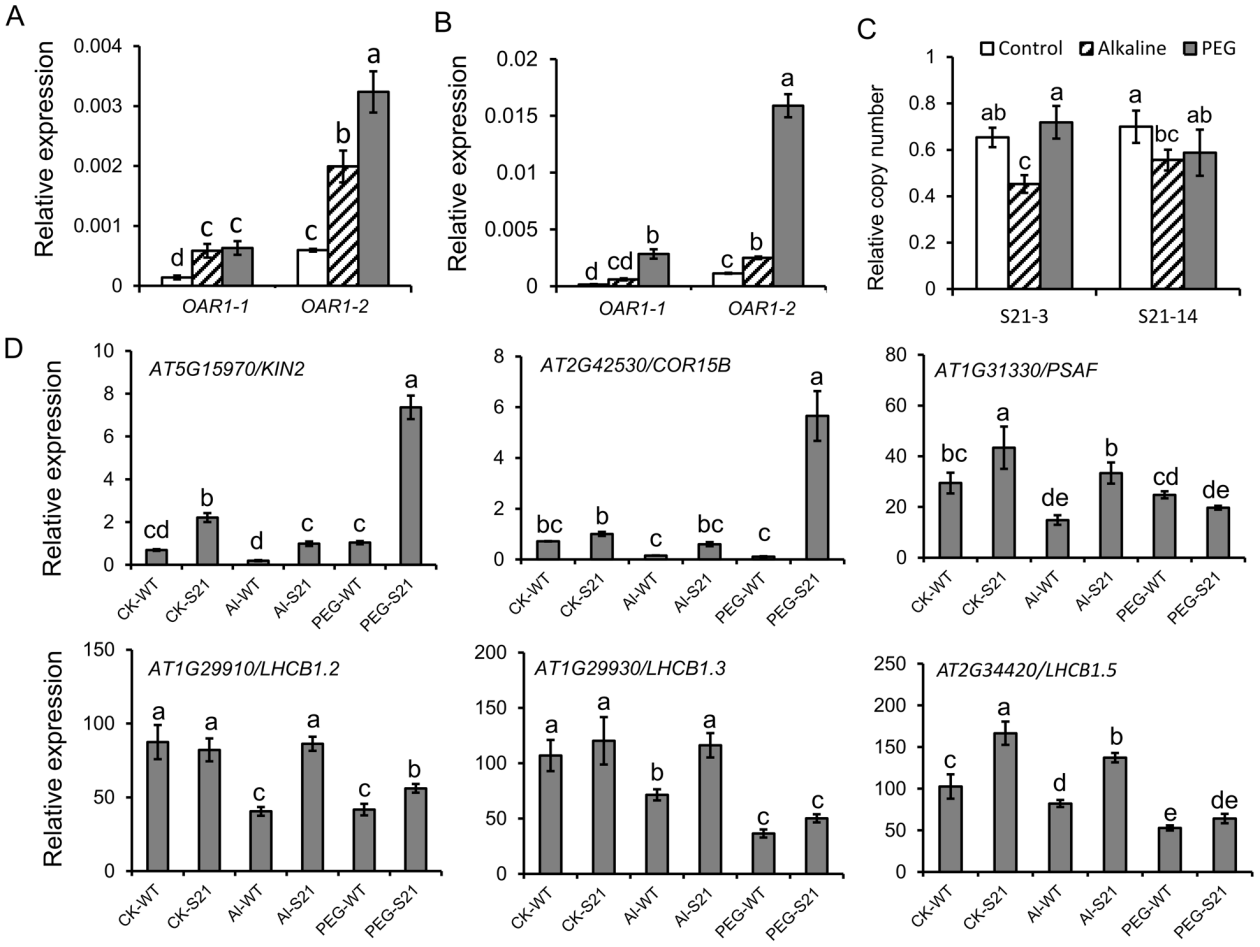
The expression and relative copy numbers of *OAR1* element, and marker gene expression in transgenic *Arabidopsis* under osmotic and alkaline stresses. (A, B) The expression of *OAR1* element in S21-3 (A) and S21-14 (B); (C)The relative copy numbers of *OAR1* element in S21-3 and S21-14; (D) The expression of stress marker gene and photosynthesis related gene in S21-3 and S21-14. Data are shown as means ± SD.

To understand the molecular mechanisms that *OAR1* may function in osmotic and alkaline resistance, we first determined if *OAR1* element was transcribed in transgenic *Arabidopsis*. Despite that there was no any known promoter sequence adjacent to S21 insert in the BIBAC vector, quantitative RT-PCR had detected high levels of two short transcripts corresponding to 0.4–0.5 kb (designated as *OAR1-2*, by primer pair of OAR1-2F and OAR1-2R) and 1.7–1.9 kb (designated as *OAR1-1*, by primer pair of OAR1-1F and OAR1-1R) regions of *OAR1* sequence in both S21-3 and S21-14 under unstressed condition and osmotic and alkaline-stressed conditions (Fig. 8A, B). No product was amplified when primers OAR1-1F and OAR1-2R were used to amplify the long transcript corresponding to 0.4–1.9 kb regions of *OAR1* sequence in transgenic plants under unstressed or stressed condition, indicating that *OAR1-1* and *OAR1-2* were either transcribed separately, or spliced from a long unstable transcript of *OAR1* in the transgenic plants. It is noticed that expression levels of *OAR1-1* and *OAR1-2* was higher in osmotic and alkaline stressed plants compared to that in unstressed plants (Fig. 8A, B).

Furthermore, relative gene copy number was examined by genomic quantitative PCR to check if the phenotype of the transgenic lines were dependent on copy number and if *OAR1* element was capable of transposition. Data indicated that the relative copy number was <1 in both S21-3 and S21-14 after normalized to NPTII gene which was co-transformed with *OAR1* within the T-DNA left and right borders in the same vector (Fig. 8C). Considering that the two transgenic lines exhibited a 3:1 ratio of survival on kanamycin in T2 generation and 100% survival on kanamycin in T3 generation, it was likely that only single copy of *OAR1* existed in the genome of S21-3 and S21-14 plants and no transposition of *OAR1* had occurred. Furthermore, the copy number of *OAR1* in S21-3 and S21-4 remained unaltered under osmotic stress but slightly decreased under alkaline stress. Because retrotransposons usually transpose in a “copy-paste” manner, the unchanged copy number indicated that *OAR1* element was not transposed in S21 transgenic plants.

### Marker gene expression in *S21* transgenic *Arabidopsis* plants

**Figure 9:**
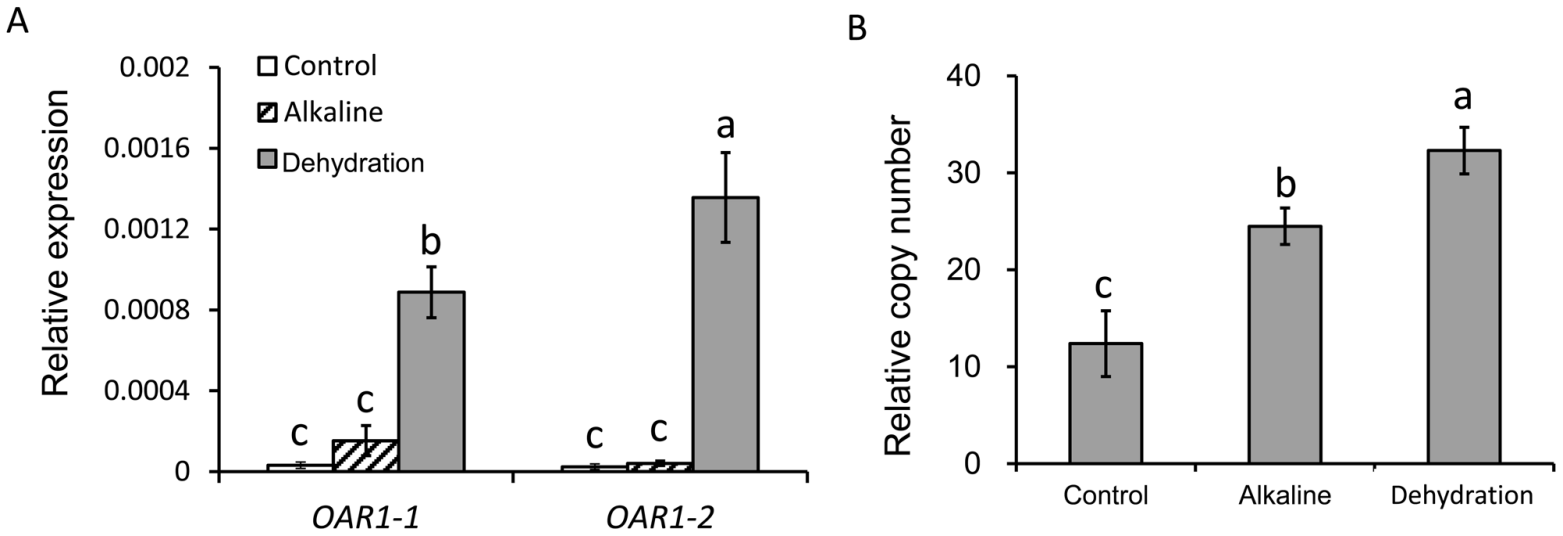
The expression and relative copy numbers of *OAR1* element in *B. hygrometrica* under osmotic and alkaline stresses. (A) The expression of *OAR1* element, *OAR1-1* and *OAR1-2* in *B. hygrometrica*; (B) relative copy number of *OAR1*. Data are shown as means ± SD.

To examine whether the insertion of *OAR1* element influenced stress responsive gene expression, several marker genes in osmotic and alkaline stress pathways were checked for expression changes, along with 4 genes related to photosynthesis. The data have shown that all test genes, with an exception of *AHA2*, increased transcription in S21-3 plants under unstressed conditions, and remained higher than that in the wild type under both osmotic and alkaline stresses (Fig. 9D). This is consistent to the observed stress tolerant phenotype and the stabilized photosynthetic apparatus in the transgenic plants.

### *OAR1* transcription and copy number in *B. hygrometrica*

To understand the molecular nature and the mechanisms that *OAR1* functions in its native host genome of *B. hygrometrica*, the expression levels of *OAR1* and the relative copy number have been determined. The results showed that *OAR1* transcription levels were increased slightly in response to alkaline and significantly under dehydration stresses in *B. hygrometrica* (Fig.9A). *B. hygrometrica* genome contained as high as >10 copy numbers of *OAR1*, and the relative copy number was increased to 2–3 fold under alkaline and PEG treatments (Fig. 9B). Considering that *OAR1* was located within a stretch of nested retroelement regions in the *B. hygrometrica* genome, as revealed by the sequence of BIBAC clone L1-4, our data indicated that *OAR1* might locate in an active retroelement cluster in its host genome.

## Discussion

In species which survive in extreme natural environments, certain adapted traits are expected to be established during evolution, to allow the species to cope with harsh conditions, such as osmotic, alkaline, and salt stresses [46]–[48]. Recent studies have shown the involvement of chromatin modification and assembly, distantly located regulatory elements, complex loci, transposable elements, small RNAs, and non*-*coding RNAs in the regulation of various plant stress responses [49], [50]. Unraveling stress*-*associated genomic regulatory mechanisms in these plants will enable future molecular manipulation of highly stress*-*tolerant crops. Limestone*-*inhabiting *B. hygrometrica* is one of the few resurrection species in the Gesneriaceae family that the vegetative tissues can survive desiccation and recover upon rehydration [7], [10]. Thus it is a good resource for isolation of functional genes and genomic elements for crop breeding to improve tolerance to drought and alkaline stresses. In this paper, we identified *OAR1*, a functional element located in a 49-kb nested LTR*-*retrotransposon fragment that conferred osmotic and alkaline stress tolerance in transgenic *Arabidopsis*, taking the advantage of BIBAC library transformation system. This finding provides the first insight into the role of nested LTR*-*retrotransposon clusters in the genome of *B. hygrometrica* in environmental adaptation of this resurrection plant.

Transformation of large genomic DNA fragments into recipient plants has been used primarily to identify genes or quantitative trait loci (QTL) in species in which map*-*based cloning is not practical, allowing for the isolation of the *FILAMENTOUS FLOWER* gene [51], [52]. This technique was soon applied for the discovery of novel genes and genomic elements from species lacking genome sequence information, genetic transformation methods, or mutation tools [21], [22]. In this paper, this laborious approach has been successfully applied to identified the 49-kb fragment that conferred stress tolerance, and herein the 2-kb *OAR1* element from *B. hygrometrica* by combination with shotgun sequencing, subcloning and phenotypic screening.

Photosynthesis has been recognized as the most sensitive process that was affected by water stress and other abiotic stresses such as salt and alkali [53]. The carbon balance, ROS homeostasis, energy generation, growth and survival of a plant under water stress depend heavily on the degree and velocity of photosynthesis decline during water depletion. Accumulating evidence demonstrated that in resurrection plants, complex mechanisms involving compatible solutes and desiccation-associated proteins such as LEAs and sHSPs, antioxidants, membrane protectants were triggered by water loss, which contribute to the stabilization of photosynthetic pigment-protein complexes and chlorophyll content, protection of photosynthetic apparatus and prevention of ROS accumulation from photosystems (PSI and PSII) [7], [10], [11], [12], [54], [55]. For example, *BhLEA1* and *BhLEA2* are two dehydration-inducible *LEA* genes from *B. hygrometrica*. When overexpressed in tobacco, they conferred improved drought tolerance, higher photosystem II activity, lower membrane permeability and more stable ribulose-bisphosphate carboxylase (large subunit), light-harvesting complex II and photosystem II extrinsic proteins under drought stress [11]. Similarly, in this paper, the transgenic plants harboring *OAR1* from *B. hygrometrica* also displayed higher survival rates, better growth, high levels of membrane integrity and photochemical efficiency, and stable expression of photosynthesis related genes and drought-induced marker genes under PEG-mediated osmotic stress.

Drought tolerance and drought avoidance are two major mechanisms in drought resistance of higher plants [56]. No difference in rate of water loss was detected between transgenic and the wild-type plants, indicating that the transformation of the *B. hygrometrica* genome fragments containing *OAR1* into *Arabidopsis* conferred plant osmotic tolerance via stabilization of photosynthetic apparatus, instead of drought avoidance.

Transposable elements can modulate gene expression and regulatory patterns in various ways, and have been described as “distributed genomic control modules” [57] at the core of regulatory networks to specific stimuli [58]. The identification of an *OAR1* element that confers osmotic and alkaline stress tolerance, isolated from native desiccation and calcarenite tolerant *B. hygrometrica* has provided the first reverse genetics evidence for the possible function of this type of retroelements in plant tolerance to abiotic stresses. It is not clear so far by what mechanisms that *OAR1* function to maintain photochemical efficiency in stress tolerance in transgenic plants. What we have known is that *OAR1* is a part of an active LTR-retrotransposon cluster, and this type of retroelement had been identified from another resurrection plant *C. plantagineum* by T-DNA activation tagging, namely *CDT-1* (*desiccation-tolerant-1*) and its homologue *CDT-2* [16], [17]. *CDT-1* could direct the synthesis of a double-stranded 21 bp short interfering RNA (siRNA), which triggered the regulatory pathway for desiccation tolerance through activation of stress-responsive genes [18]. Small-RNA coding as described for *CDT-1* present a possible analogous mechanism by which *OAR1* might regulate gene expression under stress conditions in transgenic plants.

Transcription of transposon genes without any known promoter-like sequence had been observed with retrotransposons such as *Sadhu* elements [59], [60]. In this study, short transcripts generated from *OAR1* could be detected in both transgenic *Arabidopsis* and *B. hygrometrica*, suggesting this 2-kb element itself is capable of activating its transcription. Despite that both the transposition activity and transcription of *OAR1* were activated in *B. hygrometrica* in its dry and alkaline native habitat, the unchanged relative copy numbers in osmotic and alkaline-stressed plants indicated that the 2-kb *OAR1* element alone in S21 transgenic *Arabidopsis* plants was not transposable. Thus *OAR1* may function in a transposition-independent manner to confer plant osmotic and alkaline resistance in the transgenic plant. In other words, *OAR1* element may have an impact on the expression of certain category of genes, probably via encoding short transcripts. Further investigation on the function of these elements in plant stress tolerance, will aid further understanding of the possible mechanisms which gave rise to the evolution and development of desiccation tolerance in *B. hygrometrica* (and possibly also other Gesnericeae resurrection plants) in alkaline and dry habitats.

## Supporting Information

File S1 (DOC)
