# Supplementary material for: Identification of a Retroelement from the Resurrection Plant Boea hygrometrica That Confers Osmotic and Alkaline Tolerance in Arabidopsis thaliana
Source: PLoS One. 2014 May 22;9(5):e98098. doi: 10.1371/journal.pone.0098098 (PMC4031123; doi:10.1371/journal.pone.0098098)
Supplement: File S1 — (DOC) [file pone.0098098.s001.doc]

Identification of a retroelement from the resurrection plant *Boea hygrometrica* that confers osmotic and alkaline tolerance in *Arabidopsis thaliana*

Yan Zhao, Tao Xu, Chun-Ying Shen, Shi-Xuan Chen, Guang-Hui Xu, Li-Zhen Song,, Mei-Jing Li, Li-Li Wang, Yan Zhu, Wei-Tao Lv, Zhi-Zhong Gong, Chun-Ming Liu, Xin Deng

**Supporting information File S1**

**Table S1 Primers used in this study.**

**Figure S1 Phenotype comparison of wild-type and transgenic plants harboring empty BIBAC vector and L1-4 under osmotic and alkaline stresses.**

**Figure S2 Phenotype comparison of wild-type and transgenic plants of L1-4 under alkaline (pH 9.0) stress.**

**Figure S3 Comparison of wild-type and transgenic plants of L1-4 and S21 under drought stress for 7 days.**

**Figure S4 Identification of the insertion site of the transgene in transgenic line L1-4-2 and the expression of the flanking genes, *TUA3* and *TUA5*.**

**Figure S5 Determination of the insert size of BIBAC clone L1-4.**

**Figure S6 Stability analysis of the plasmid DNA in L1-4 BIBAC clone in *Agrobacterium tumefaciens* strain GV3101.**

**Figure S7 Phenotype of the transgenic plants over-expressing *OAR1* under osmotic and alkaline stress conditions.**

**Figure S8 Phenotype comparison of wild-type and transgenic plants harboring S32 and S35 under osmotic and alkaline stresses.**

**Figure S9 Relative water content determination and stomatal observation of the wild-type and S21-3 transgenic plants under osmotic stress.**

**Table S1 Primers used in this article.**

| **Primers** | **Sequence** | **Site** |
| --- | --- | --- |
| M13F-47 | CGCCAGGGTTTTCCCAGTCACGAC | vector |
| 1-4R | ACGCCCGTTCTACATCAATAC | -49076 |
| 1kF | ATGGACAGACGAGTGTGAGT | +159 |
| 1kR | AATCGGGAAGTAAATCGTGG | -48173 |
| 3kF | ACGAGCGGGTGGTACTATGT | +2602 |
| 3kR | CAACAGAACAGAACTGGGAG | -45953 |
| 4kF | CTTCTTGGGCTACTTTGGTCT | +4058 |
| 4kR | ACCTTGTGCAACCAACTACAC | -44567 |
| 5kF | GCACAGGCGTTGGTTGACTT | +5224 |
| 5kR | GCGGCATCTACGAATACACTCA | -42354 |
| 14kF | GATTTGAGTTGCCAGTTGATAG | +14172 |
| 14kR | TTACTCTTCGTTCGCTCTTGG | -34029 |
| 29kF | TTCTACCCCTGGAGGTGTGA | +29561 |
| 29kR | ATTCTCAGGTGCTCCTTATG | -19412 |
| 42kF | ACTCTACCAAAGACAATGCG | +42869 |
| 42kR | ATTTGAGTCGCCAGTTGATA | -5408 |
| 44kF | AAATGTCTCGGTGAACAGTAG | +44589 |
| 44kR | GTCCCTTTGAGATTATTGAGC | -3588 |
| 46kF | CCAAAAGCGAGAAGTAAACC | +46348 |
| 46kR | TGTGTGCTCTGATGCTTCT | -2023 |
| 47kF | TCGCACTCATCAGTCCAAAC | +47444 |
| M13R-48 | AGCGGATAACAATTTCACACAGGA | vector |
| TUA3F | AATCAACTACCAACCTCCAA |  |
| TUA3R | CCTCCATTCCTTCACCAACG |  |
| TUA5F | GCAGAGGTGTTCTCACGGAT |  |
| TUA5R | GAGAAAAGGTGTCAATAGTC |  |
| OAR1-1F | TCGCACTCATCAGTCCAAAC | +47444 |
| OAR1-1R | CTGTAGATCCGAGCAAGGTC | -1776 |
| OAR1-2F | CTCGCAGGATAATCATAAATAG | | +48818 | | --- | |
| OAR1-2R | CGAATGCCTTGACAGGAGAT | -485 |
| AT5G15970F | CAAAACACACATCAAAAACG |  |
| AT5G15970R | TACTCTTTCCCGCCTGTTGC |  |
| AT2G42530F | GGCGATGTCTTTATCAGGAG |  |
| AT2G42530R | AGGATGTTGCCGTCACTTTT |  |
| AT1G31330F | AGGACTCAAAACAGTTCGCT |  |
| AT1G31330R | GTAAACCGTCTGACCCGCAT |  |
| AT1G29910F | AATGAGGAAGACTGTTGCCA |  |
| AT1G29910R | CGGTGTCCCATCCGTAGTCT |  |
| AT1G29930F | GCTCTCTCCTCCCCTGCCTT |  |
| AT1G29930R | ACGGTTCCTTGCGAATGTCT |  |
| AT2G34420F | CGGTGACTACGGATGGGACA |  |
| AT2G34420R | CCAATCCTCCGTCGCTGAA |  |
| 18S-F | CTTAGTTGGTGGAGCGATTTG |  |
| 18S-R | CCTGTTATTGCCTCAAACTTCC |  |
| Actin2F | TTCCCGTTCTGCGGTAGTGG |  |
| Actin2R | CCGGTATTGTGCTCGATTCTG |  |
| SP1 | GCCGAGTTGACAGACTGCCTA |  |
| SP2 | TGGGAATGGCGAAATCAAGGC |  |
| SP3 | AATAACGCTGCGGACATCTAC |  |
| AD1 | NTCGASTWTSGWGTT |  |

N=A/C/G/T；S=G/C；W=A/T.

**Figure S1**


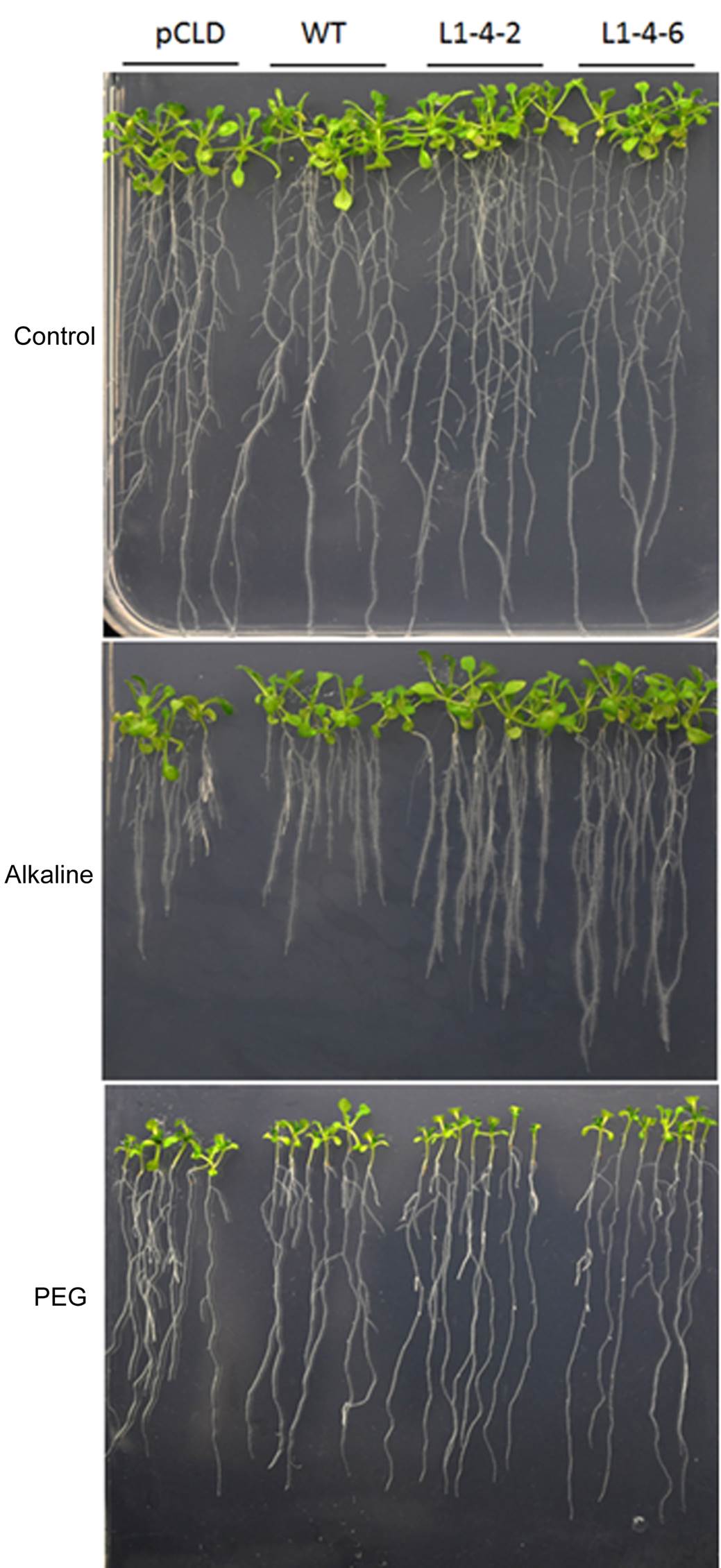


**Figure S1 Phenotype comparison of wild-type and transgenic plants harboring empty BIBAC vector and L1-4 under osmotic and alkaline stresses.** Seedlings of the wild-type and transgenic plants of L1-4 were transferred onto 1/2 strength MS agar plates adjusted to pH 5.6 (Control), pH 8.5 (Alkaline), and onto 1/2 strength MS agar plates soaked by 25% PEG 8000 (PEG).

**Figure S2**


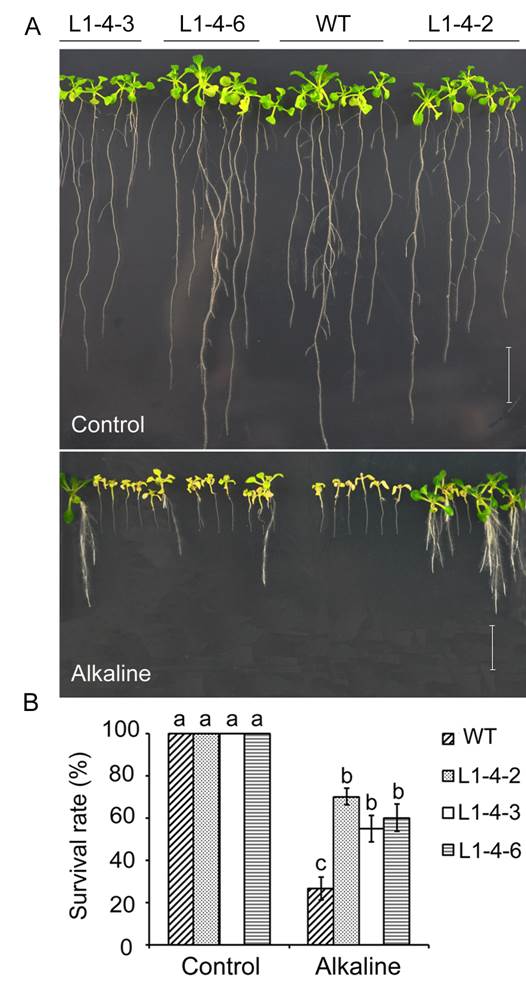


**Figure S2 Phenotype comparison of wild-type and transgenic plants of L1-4 under alkaline (pH 9.0) stress.** (A) Seedlings grown on agar plates containing 1/2 MS with pH5.6 (Control) and pH 9.0 (Alkaline) for 14 d. (B) Survival rate of wild*-*type plants and transgenic lines of L1-4 on alkaline media (pH 9.0). Plants with chlorina cotyledons and undeveloped true leaves were considered as died. n = 18, Bar = 1cm. Data are shown as means ± SD.

**Figure S3**


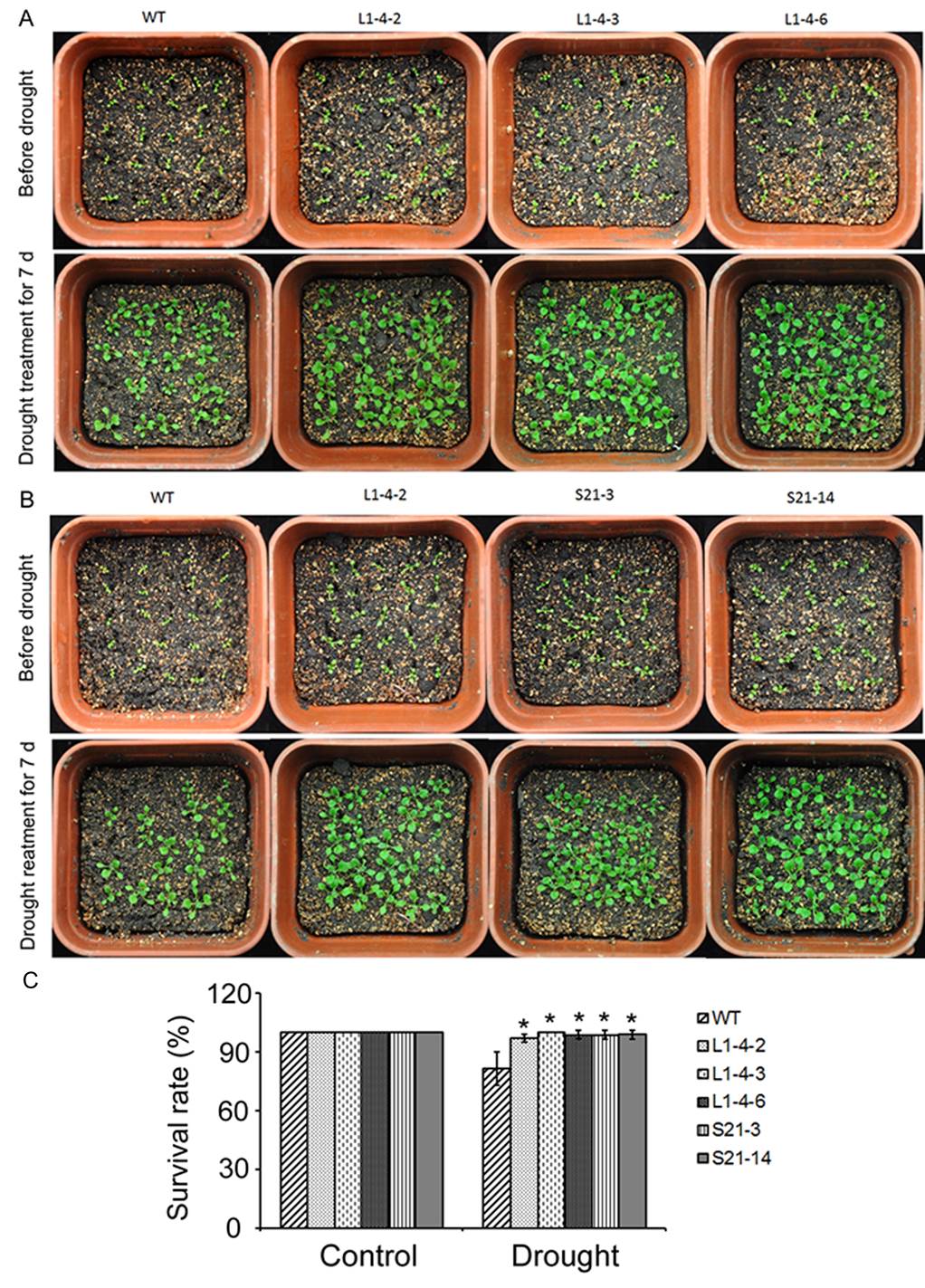


**Figure S3 Comparison of wild-type and transgenic plants of L1-4 and S21 under drought stress for 7 days.** Comparison of wild-type and transgenic plants of L1-4 (A), wild-type and transgenic plants of S21 (B) under drought stress. (C) Survival rate of wild-type and transgenic plants of L1-4 and S21 under drought stress for 7 days. Three replicates of 25 seedlings were tested in each treatment. Data are shown as means ± SD.

**Figure S4**


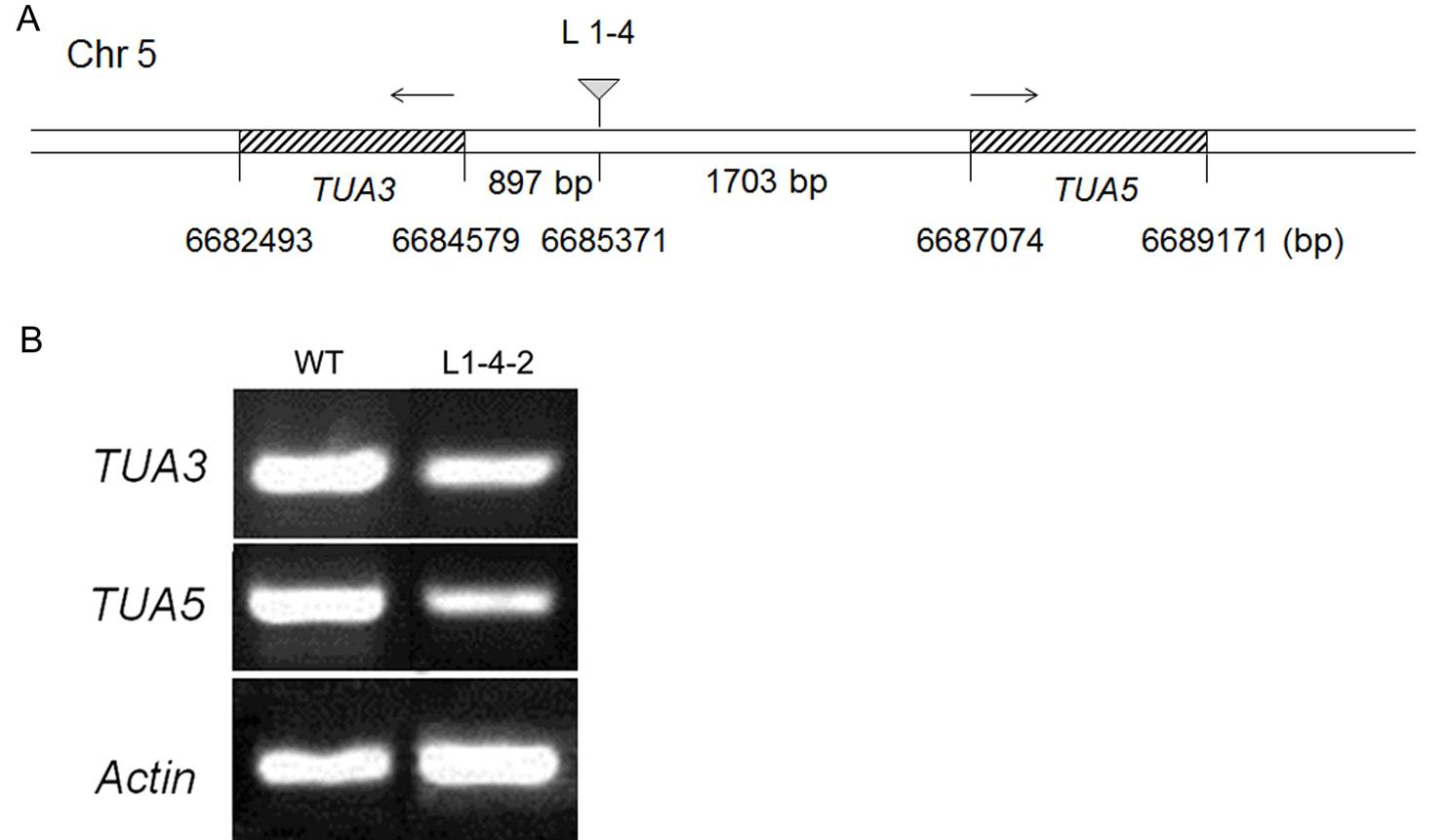


**Figure S4 Identification of the insertion site of the transgene in transgenic line L1-4-2 and the expression of the flanking genes, *TUA3* and *TUA5*.** (A) Identification of the insertion site of the transgene in transgenic line L1-4-2. [Genomic](app:ds:  genome) DNA of transgenic line L1-4-2 was extracted and used as the template for Tail-PCR. The inverted triangle indicates the insertion site of L1-4 DNA in chromosome 5 of *Arabidopsis*; the shadowed box indicates tubulin alpha. (B) RT-PCR of *TUA3* and *TUA5* under unstressed condition.

**Figure S5**


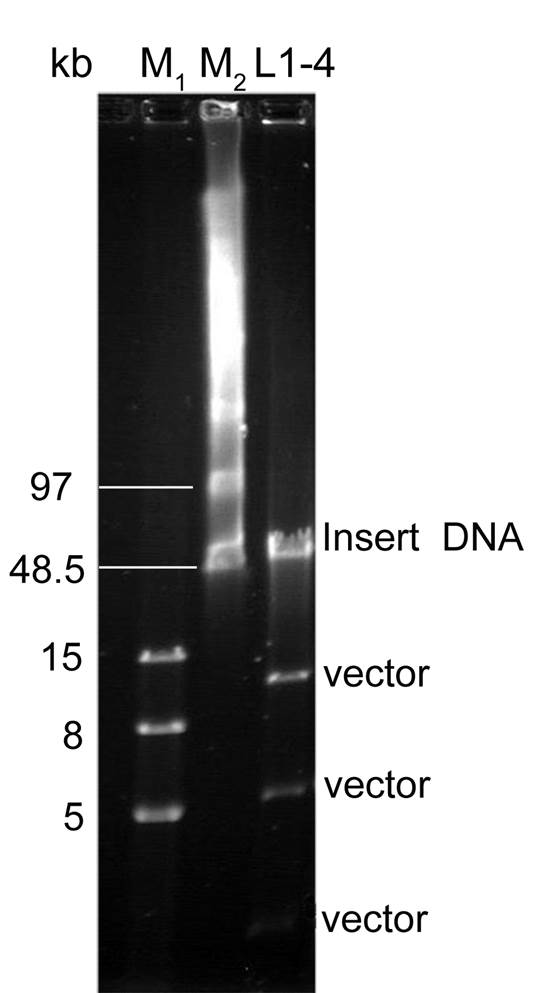


**Figure S5 Determination of the insert size of BIBAC clone L1-4.** Plasmid DNA of L1-4 was digested with *Not*I and separated by PFGE, stained with ethidium bromide and photographed. M_1_, marker with band sizes of 3, 5, and 8 kb respectively; M_2_, Lamda ladder PFG marker with band sizes of 48.5, 97, 145, and 194 kb, respectively.

**Figure S6**


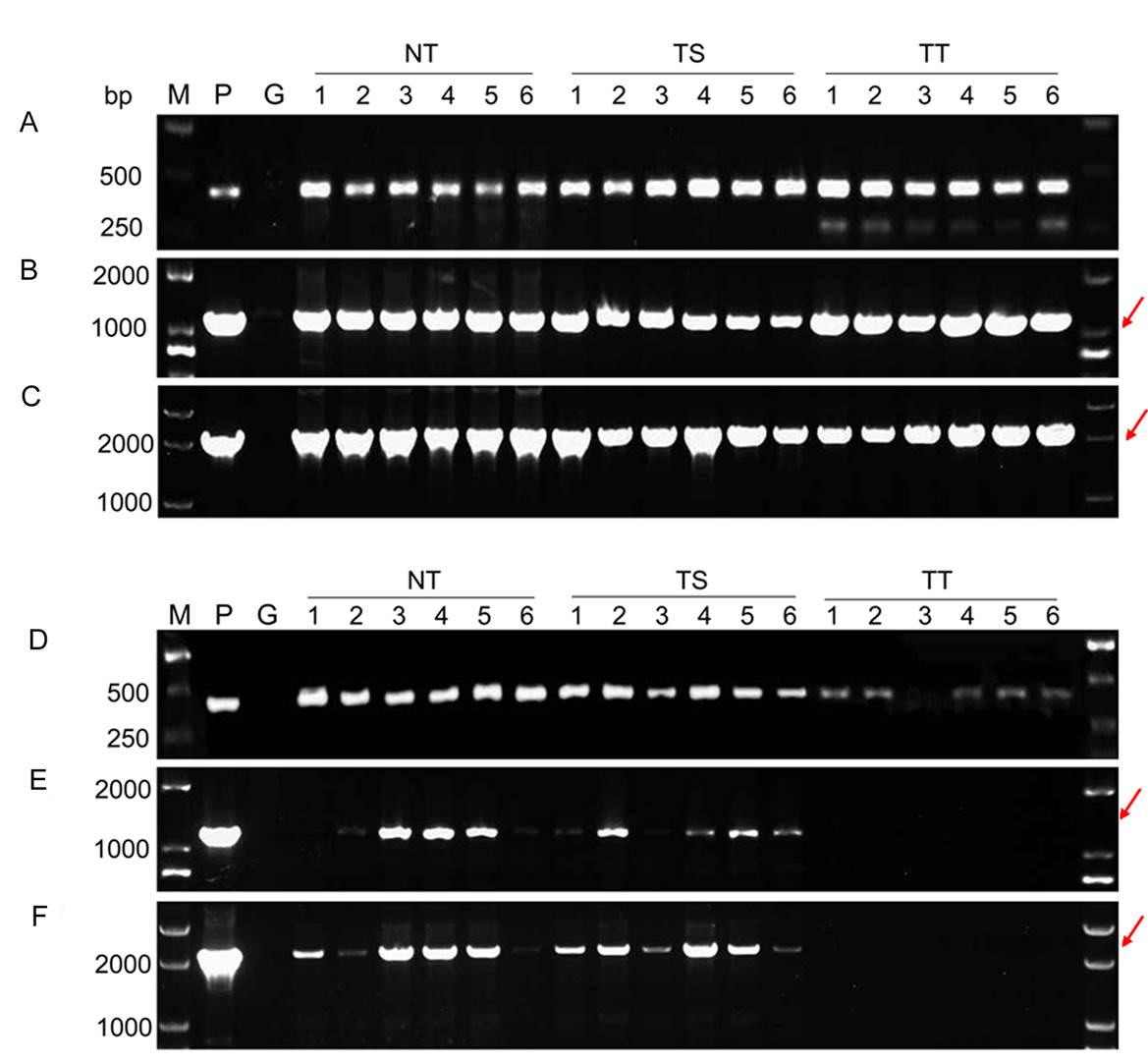


**Figure S6 Stability analysis of the plasmid DNA in L1-4 BIBAC clone in *Agrobacterium tumefaciens* strain GV3101.** Six clones (1, 2, 3, 4, 5, and 6) of *Agrobacterium* that were newly transformed (NT), stored in -80°C for six months (TS) and 3 years after transformation (TT), respectively, were randomly selected and analyzed by PCR in the first culture (A-C) and the fifth culture (D-F) using four pairs of L1-4-specific primers for amplification of the 425 bp left end fragment (A, D), 1188 bp middle part fragment (B, E) and 2115 bp right end fragment (C, F). M, marker; P, plasmid of 1-4; G, GV3101.

**Figure S7**


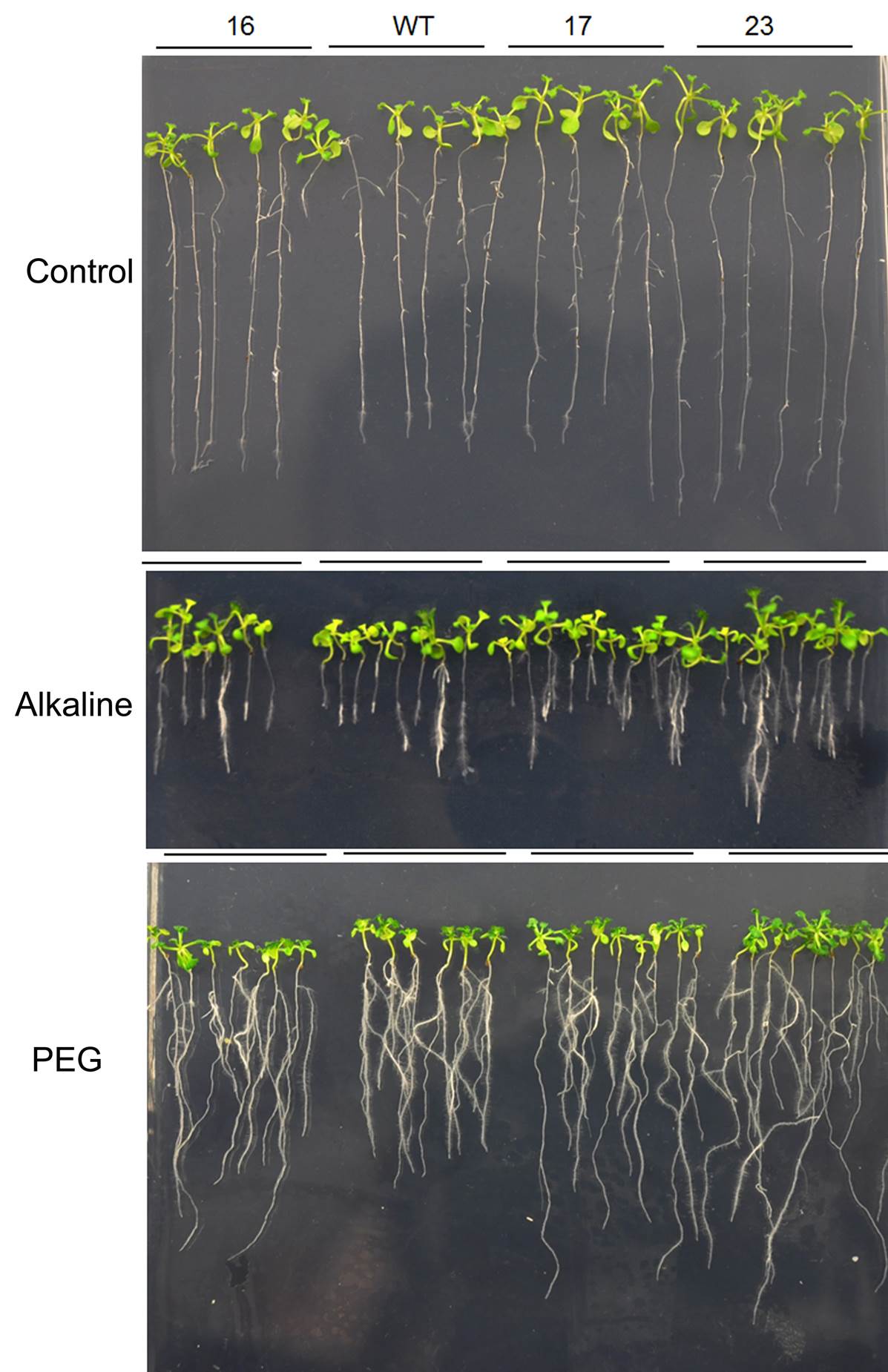


**Figure S7 Phenotype of the transgenic plants over-expressing *OAR1* under osmotic and alkaline stress conditions.** Seedlings grown on 1/2 MS agar plates adjusted to pH 5.6 (Control) and pH 8.5 (Alkaline), and 1/2 MS agar plates saturated with 40% PEG 8000 (PEG). 16, 17, and 23 were random transgenic plants over-expressing *OAR1*.

**Figure S8**


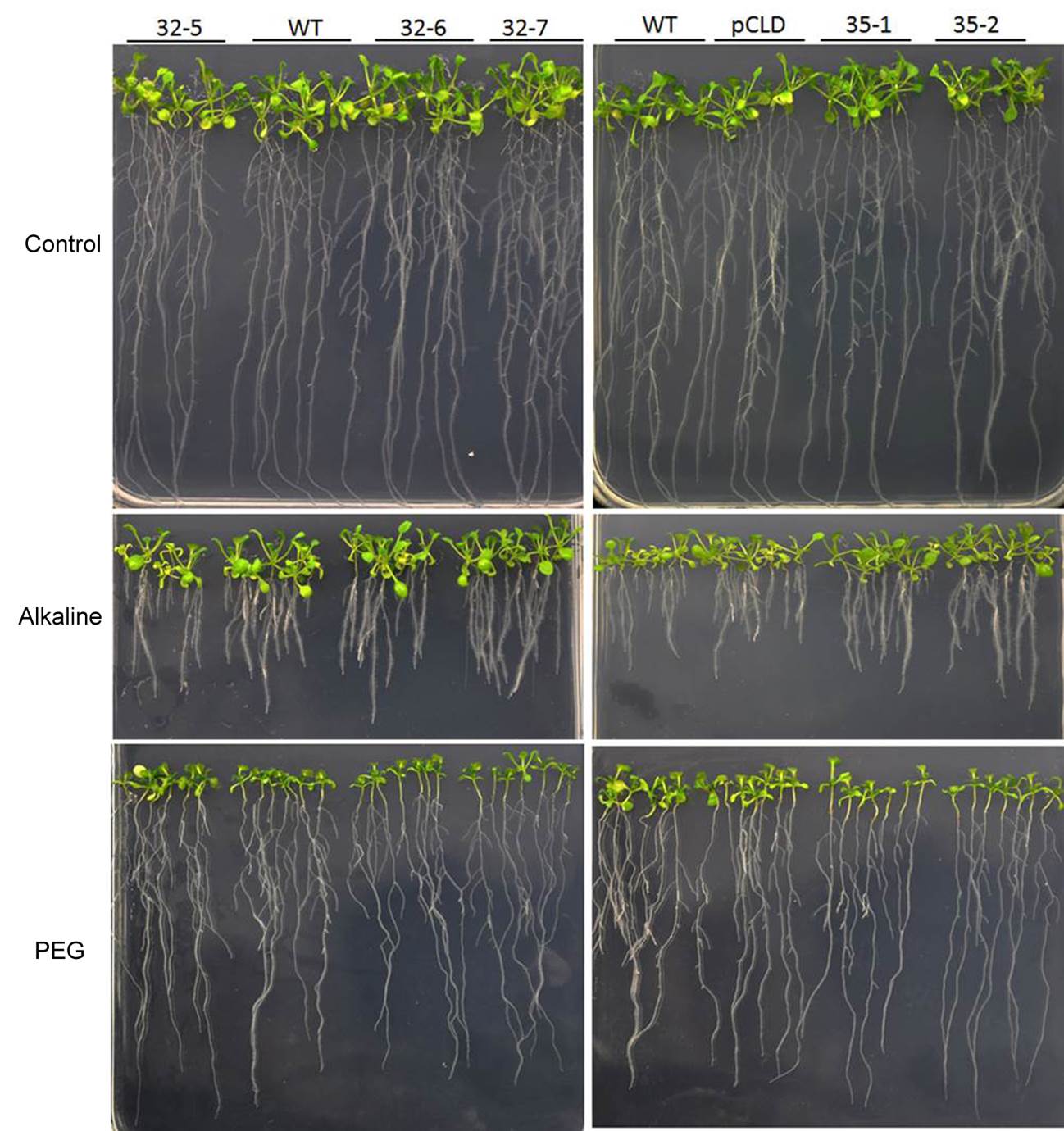


**Figure S8 Phenotype comparison of wild-type and transgenic plants harboring S32 and S35 under osmotic and alkaline stresses.** Phenotype comparison of the wild-type and transgenic plants of S32 and S35 were transferred onto 1/2 strength MS agar plates adjusted to pH 5.6 (Control), and onto plates adjusted to pH 8.5 with potassium hydroxide (Alkaline), and onto 1/2 strength MS agar plates soaked by 25% PEG 8000 (PEG). 32-5, 32-6 and 32-7, independent transgenic lines of S32; 35-1 and 35-2, independent transgenic lines of S35.

**Figure S9**


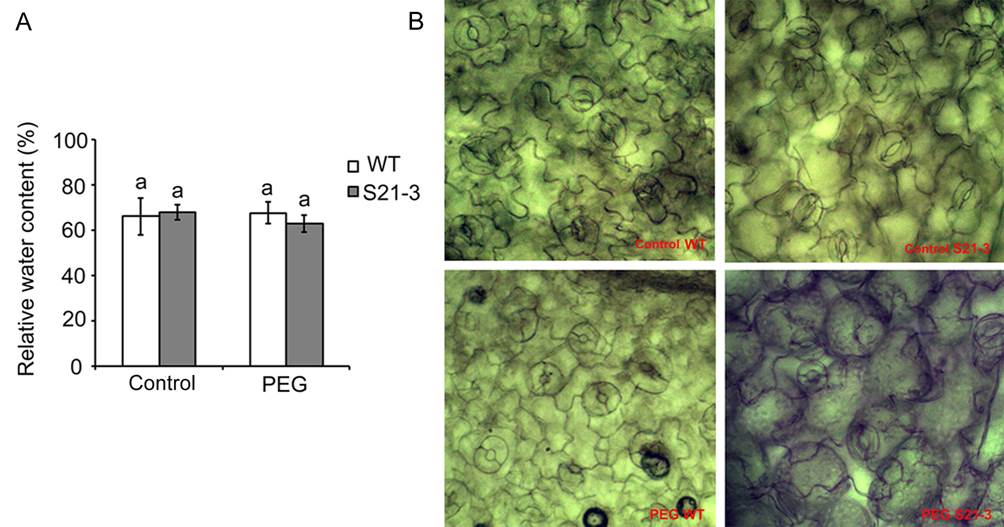


**Figure S9 Relative water content determination and stomatal observation of the wild-type and S21-3 transgenic plants under osmotic stress.** Seedlings were grown on 1/2 MS agar plates adjusted to pH 5.6 (Control) and 1/2 MS agar plates saturated with 40% PEG 8000 for 2 weeks prior to measurement. (A) Relative water content. RWC = (FW- DW) / (TW - DW) × 100%, FW, fresh weight; DW, dry weight; TW, turgid weight. (B) Stomatal observation. Leaves were fixed with absolute ethyl alcohol for 2–3 min. Stomata were observed and recorded using light microscopy (B204LED, China).
